# BMP-Binding Polysulfonate Brushes to Control Growth Factor Presentation and Regulate Matrix Remodelling

**DOI:** 10.1021/acsami.4c05139

**Published:** 2024-07-29

**Authors:** Metzli Hernandez Marchena, Elisa Lambert, Bojana Bogdanović, Fauzia Quadir, Carlos E. Neri-Cruz, Jiajun Luo, Clemence Nadal, Elisa Migliorini, Julien E. Gautrot

**Affiliations:** † School of Engineering and Materials Science, Queen Mary University of London, Mile End Road, London E1 4NS, U.K.; ‡ University Grenoble Alpes, INSERM, CEA, CNRS, U1292 Biosanté, EMR 5000, 17 Av des Martyrs, Grenoble 38000, France

**Keywords:** BMP2, polymer brush, ATRP, biomimetic, sulfonate

## Abstract

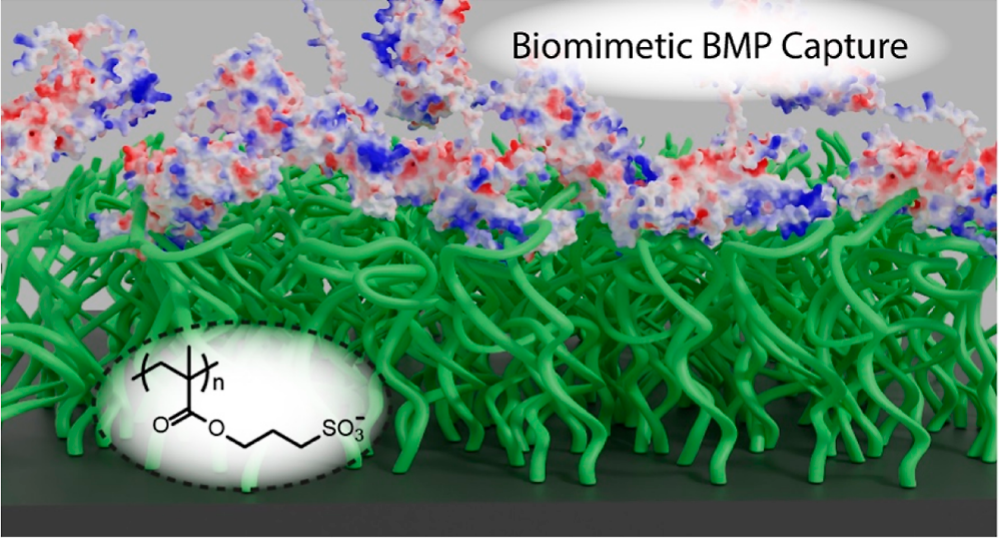

Bone morphogenetic proteins (BMPs) are important targets to incorporate in biomaterial scaffolds to orchestrate tissue repair. Glycosaminoglycans (GAGs) such as heparin allow the capture of BMPs and their retention at the surface of biomaterials at safe concentrations. Although heparin has strong affinities for BMP2 and BMP4, two important types of growth factors regulating bone and tissue repair, it remains difficult to embed stably at the surface of a broad range of biomaterials and degrades rapidly in vitro and in vivo. In this report, biomimetic poly(sulfopropyl methacrylate) (PSPMA) brushes are proposed as sulfated GAG mimetic interfaces for the stable capture of BMPs. The growth of PSPMA brushes via a surface-initiated activator regenerated by electron transfer polymerization is investigated via ellipsometry, prior to characterization of swelling and surface chemistry via X-ray photoelectron spectroscopy and Fourier transform infrared. The capacity of PSPMA brushes to bind BMP2 and BMP4 is then characterized via surface plasmon resonance. BMP2 is found to anchor particularly stably and at high density at the surface of PSPMA brushes, and a strong impact of the brush architecture on binding capacity is observed. These results are further confirmed using a quartz crystal microbalance with dissipation monitoring, providing some insights into the mode of adsorption of BMPs at the surface of PSPMA brushes. Primary adsorption of BMP2, with relatively little infiltration, is observed on thick dense brushes, implying that this growth factor should be accessible for further binding of corresponding cell membrane receptors. Finally, to demonstrate the impact of PSPMA brushes for BMP2 capture, dermal fibroblasts were then cultured at the surface of functionalized PSPMA brushes. The presence of BMP2 and the architecture of the brush are found to have a significant impact on matrix deposition at the corresponding interfaces. Therefore, PSPMA brushes emerge as attractive coatings for scaffold engineering and stable capture of BMP2 for regenerative medicine applications.

## Introduction

Bone morphogenetic proteins (BMPs) are growth factors belonging to the transforming growth factor-beta superfamily. Over 15 μ_B_Ps have been identified in mammals^1^ and play important roles in tissue homeostasis, cell differentiation, and cell reprogramming, in a wide range of different cell types (bone, cartilage, endothelium, etc.).^2^ BMP 2 has been identified in the 1970s as an essential molecule for de novo bone formation in adult animals.^3,4^ Owing to its osteogenic potential, the clinical use of recombinant human BMP2 has been approved in 2002 by the Food and Drug Administration and validated by the European Medicines Agencies. Subsequently, further biological functions of BMP2 were identified.^5^ BMP4 presents a structure 80% homologous to that of BMP2.^6^ Several studies have similarities in the properties of BMP2 and BMP4, on physiological and pathological processes.^7−9^ In terms of molecular interactions, it is known that BMP2 and BMP4 interact with BMP type-IA (BMPR-IA) (also known as ALK3), BMPR-IB (ALK3), and BMP type-II receptors, although with different affinities.^10^ In particular, BMP2 presents higher affinities for both type-I and type-II receptors with respect to BMP4.

After their synthesis and secretion, BMP2 and 4 interact with proteoglycans, which are present both at the cell surface and within the extracellular matrix. In particular, both BMP2 and BMP4 present a heparin binding site at their N-terminal end. Therefore, both growth factors bind to heparan sulfate but with different binding kinetics.^11,12^ Their binding to collagen is also different. It has indeed been demonstrated that the N-terminal prodomain of collagen type IIA binds BMP2,^13^ whereas the C-terminal part of collagen IV binds BMP4,^14^ suggesting that in nature BMP2 and BMP4 are differently distributed in tissues since they interact differently to extracellular matrix components.

A broad range of strategies have been proposed for the incorporation of growth factors and BMPs into biomaterials and their release or presentation, for tissue engineering and regenerative medicine applications.^15^ The loading and release of high doses of BMP2 by/from implants were found to be directly linked to tumor formation.^16^ However, more recently, the stable adsorption of BMP2 and its surface activity (without release) were effective in stimulating the SMAD pathway and downstream signaling mechanisms and cellular phenotype regulation, at BMP2 doses that are orders of magnitude below.^17^ Therefore, stable surface adsorption appears as a translatable strategy to harness BMP biology for tissue regeneration. Hence, self-assembled monolayers, hydrogels, and polyelectrolyte multilayers can capture or allow the coupling of BMP2 and other growth factors and cytokines, for example, using hyaluronic acid backbones or histidine tags, to regulate cell adhesion and motility, neural stem cell maintenance, as well as osteoblast differentiation and bone regeneration.^18−21^ While these approaches are not directly aiming at mimicking the binding of growth factors by glycosaminoglycans (GAGs), they were found to be effective and allow the control of a broad range of other parameters, such as matrix stiffness, patterning, and the presentation of other ligands for cell membrane receptors, such as integrins.^22,23^ Sulfated GAGs such as heparin, heparin sulfate, and chondroitin sulfate, which have the capacity to bind a range of growth factors, have been introduced in a broad range of biomaterial designs, including for the coating of fibers, the backbone of hydrogels, the formation of polyelectrolyte multilayers, or in engineered monolayers, for the capture of growth factors including basic fibroblast growth factor, TGF-β, and vascular endothelial growth factor.^24−30^

Although several studies suggested the use of GAG-based biomaterials for tissue engineering to control inflammation, inflammatory cells can release proteases and glycanases that mediate degradation of collagens and GAGs in the extracellular matrix. In addition, natural polysaccharides present in the extracellular matrix and used to engineer GAG-based biomaterials have a complex molecular structure and can be sulfated at different positions, giving rise to a vast number of possible combinations of sulfated motifs. To avoid the degradation of GAGs in biomaterials, several strategies have proposed GAG-mimetic designs.^31^ The generation of low molecular weight heparin-poly(ethylene glycol) conjugates was proposed for the design of hydrogels, cross-linked by heparin-binding peptide conjugates, and enabling the capture of the basic fibroblast growth factor and vascular endothelial growth factor.^32,33^ Similarly, the design of a range of sulfated maltose oligo-dendrimers (e.g., presenting 4 arms each displaying sulfated dimers) allowed the identification of a particularly strong heparin biomimetic candidate to enhance BMP2 activity in vitro and bone regeneration, although this required multistep synthesis.^34^ Various sulfated vinyl saccharides have also been proposed as heparin-mimicking polymers, for the binding of growth factors and to prevent amyloid β aggregation.^35−37^ Another strategy proposed for the capture of heparin-binding growth factors was based on the design of copolymers featuring styrenesulfonate residues. This enabled the design of copolymers allowing the stabilization of fibroblast growth factor 2 and promoted binding of the corresponding receptor [through a poly(vinyl sulfonate) block].^38^ Similarly, nanopatterns of poly(styrenesulfonate) (PSS) and RGD peptides allowed the capture of the basic fibroblast growth factor and the regulation of cell adhesion.^39,40^

Polymer brushes present interesting features for the biofunctionalization of biomaterial surfaces, owing to the unique control of their molecular structure, physicochemical properties that can be achieved through monomer selection, and architectural design.^41−44^ Hence, some polymer brushes display unique protein-resistant properties allowing resistance even in complex concentrated physiological fluids for biosensing applications.^45−47^ Other brushes enable the tethering of cell adhesive peptides and protein fragments to regulate cell spreading, motility, and endothelialization or osseointegration.^48−50^ Polymer brushes have also been extensively applied to the formation of cell sheets for regenerative medicine^51,52^ and for the capture of nucleic acid materials for gene delivery applications.^53−55^

Although the adsorption of proteins such as albumin, lysozyme, fibrinogen, and extracellular matrix components such as fibronectin and collagen has been widely studied,^56,57^ relatively few examples of growth factor immobilization on polymer brushes have been reported. For example, poly(acrylic acid) brushes were functionalized with hepatocyte growth factor and basic growth factor via physisorption and EDC/NHS coupling, for the culture and endoderm commitment of mouse embryonic stem cells.^58^ Similarly, the vascular endothelial growth factor was coupled to pegylated polyurethanes, to promote endothelialization.^59^ TGF-β was coupled to poly(ethylene glycol) brushes decorating microparticles through azide-mediated Staudinger ligation, using a grafting to approach, to modulate T-cell activation.^60^ BMP2 was coupled to poly(glycidyl methacrylate) and poly(oligoethylene glycolmethacrylate) brushes, to promote osteodifferentiation and bone regeneration.^61,62^ Heparin was also coupled to grafted copolymers of poly(*N*-isopropylacrylamide-*co*-2-carboxyisopropylacrylamide), to enable the capture of the basic fibroblast growth factor and heparin binding growth factor, for the culture of hepatocytes and hepatocyte sheets.^63,64^ Finally, sulfonated brushes such as PSS and sulfonated poly(3-*O*-methacryloyl-1,2:5,6-di-*O*-isopropylidene-d-glucofuranose) have also been proposed for the capture of monocyte chemoattractant protein-1 and the inhibition of coagulation.^65,66^

Despite their capacity to mimic the sulfate chemistry of heparin, sulfonated polymer brushes such as PSS and poly(sulfopropyl methacrylate) (PSPMA) have not been explored for the direct capture of BMPs and other growth factors. Whereas PSS has been applied to the captures of proteins,^67,68^ PSPMA brushes have received less attention for such applications and were investigated primarily as bacterial repellent or bactericidal coating.^69,70^ In this project, the ability of PSPMA to capture BMPs (specifically, BMP2 and BMP4) was investigated. The growth of PSPMA brushes through an activator regenerated by electron transfer (ARGET) mechanism was examined first, using ellipsometry, to enable the generation of brushes in ambient conditions, only requiring initial degassing of polymerization solutions. PSPMA brushes with controlled architecture (density of initiator and thickness) are demonstrated, and some of the physicochemical properties (surface chemistry and solution swelling) are characterized. The capture of BMP2 and BMP4 and the impact of PSPMA architecture on this process are then studied using surface plasmon resonance (SPR) and quartz crystal microbalance with dissipation monitoring (QCM-D). Finally, the formation of fibroblast monolayers at resulting BMP-2-functionalized surfaces and the ability of these cells to derive a mature extracellular matrix at corresponding interfaces are investigated.

## Materials and Methods

### Materials and Reagents

l-ascorbic acid, copper(I) chloride (Cu(I)Cl), copper(II) bromide (Cu(II)Br_2_), 2,2′-bipyridyl (bipy), anhydrous methanol, anhydrous toluene, triethylamine (Et_3_N), trimethoxy(propyl)silane, potassium chloride, sulfopropyl methacrylate potassium salt, and 1-undecanethiol (98%) were purchased from Sigma-Aldrich. All chemicals and solvents were of analytical grade unless otherwise stated. Cu(I)Cl was kept under vacuum until used. Silicon wafers (100 mm diameter, ⟨100⟩ orientation, polished on one side/reverse etched) were purchased from PI-KEM Ltd. Gold substrates were produced by evaporation deposition (200 nm gold/20 nm chromium) on silicon wafers. Silicon and gold substrates were cleaned in a Henniker Plasma Cleanser (HPT-200, air plasma) for 5 min. The silane initiator, (3-trimethoxysilyl)propyl 2-bromo-2-methylpropionate, was purchased from Gelest. The thiol initiator, ω-mercaptoundecyl bromoisobutyrate, was synthesized according to the literature.^71,72^ SPR chips (10 × 12 × 0.3 mm) were purchased from Ssens. BMPs: Recombinant human BMP-4 protein (314-BP) and recombinant human/mouse/rat BMP-2 protein (355-BM) were obtained from Bio-Techne, carrier free.

### Materials and Reagents for Cell Culture and Characterization

Penicillin–streptomycin (PS, catalog no. P4333), fetal bovine serum (FBS, catalog no. F9665), phosphate-buffered saline (PBS) (Sigma-Aldrich, catalog no. D1408), trypsin–EDTA (catalog no. T4049), glutaraldehyde solution (catalog no. G5882), ascorbic acid (catalog no. A92902), l-proline (catalog no. P0380), *trans*-4-hydroxy-l-proline (catalog no. H54409), FBS (catalog no. HT5011-1CS), Mowiol 4–88 (catalog no. 81381), bovine serum albumin (BSA, catalog no. A8022), and 4′, 6-diamidino-2-phenylindole (DAPI) were from Sigma-Aldrich and used as received. l-glutamine (200 mM, catalog no. 25030149) was obtained from Gibco. Gelatin (catalog no. 214340; 25% w/v) was from BD Difco. Glycine (catalog no. 444495D) was from VWR. Triton X-100 (catalog no. BP151-500) and phalloidin Alexa Fluor 555 were from Thermo Fisher Scientific. Primary antibodies: anticollagen I (Abcam, ab90395) and antifibronectin (Sigma-Aldrich, catalog no. F3648). Secondary antibodies: Alexa Fluor 488 goat antimouse and Alexa Fluor 555 donkey antirabbit antibodies were from Thermo Fisher Scientific.

### Initiator Deposition

ATRP initiators were deposited on silicon or gold substrates. For the deposition on silicon, substrates (freshly treated with air plasma) were immersed in a solution of toluene (30 mL), silane initiator (30 μL), and Et3N (50 μL), overnight. Deposition on gold was carried out in the same way but in a thiol initiator solution (5 mM, in ethanol). For low density brushes, trimethoxypropylsilane or undecanethiol (for silicon and gold substrates, respectively) were used as unreactive silane/thiol at 19:1 with respect to the initiator. Upon deposition, substrates were washed with ethanol and dried with a N_2_ stream.

### Polymer Brush Growth

For ARGET-ATRP of PSPMA, a solution of CuBr_2_ (7 mg, 0.03 mmol), bpy (46.85 mg, 0.3 mmol), and SPMA (6.65 g, 27 mmol) in 10 mL of 1:1H_2_O/EtOH was degassed via Ar bubbling for 30 min. Ascorbic acid (80 mg, 0.45 mmol) and KCl (33.3 mg, 0.28 mmol) were then added and further degassed for 15 min. Substrates were placed in a 24-multiwell plate prior to injection of 1 mL polymerization solution. The polymerization reaction was left to proceed without use of inert atmosphere and stopped at desired time points (from 0.5 to 120 min) through dilution with deionized water. Brush-functionalized substrates were washed with ethanol, dried with a N_2_ stream, and characterized by ellipsometry.

For ATRP of PSPMA, ascorbic acid was replaced with CuCl (29.7 mg, 0.3 mmol). The 24-multiwell plate was placed inside of an in-house-built sealed chamber and purged with argon for 10 min. 1 mL of polymerization solution was injected, and the reaction was stopped at different time points through dilution with deionized water. Brush-functionalized substrates were washed with ethanol, dried with a N_2_ stream, and characterized by ellipsometry.

### Ellipsometry

Brush thicknesses were characterized by spectroscopic ellipsometry with an α-SE instrument (J. A. Woollam) at an incidence angle of 70°, at multiple wavelengths (380–900 nm spectrum). The dry thickness of PSPMA brushes was first measured in air, and then substrates were transferred to a liquid cell (fitted with quartz windows normal to the light beam path) and left to equilibrate for 15 min, to study the swelling in deionized water and PBS. All measurements were carried out in triplicate, at room temperature. Psi and delta spectroscopic traces were extracted and fitted against a simple native oxide/Cauchy model in CompleteEASE (J.A. Woollam). Swelling factors were calculated as the ratio of the swollen/dry thickness.

### Scanning Electron Microscopy

Samples were coated with gold for 45 s before being imaged using a FEI Inspect F scanning electron microscope operated at 10 kV. A spot size of 3 and an aperture of 30 mm were used. Five areas were analyzed at different magnifications (200×–40,000×).

### Atomic Force Microscopy

Atomic force microscopy (AFM) was used to scan surfaces, in semicontact mode, and the row pictures were corrected with a second-order function using Gwyddion software. Noncontact NSG01 cantilevers from NT-MDT were used (force constant = 1.45–15.1 N/m and resonant frequency = 87–230 kHz).

### Surface Plasmon Resonance

Thiol-functionalized SPR chips were used to generate PSPMA brushes of 10 and 30 nm, high and low density, following the same process than for silicon/gold substrates. SPR assays were carried out in PBS on a Biacore 1K+ (Cytiva) instrument in triplicate. The flow rate of injections/running buffer was maintained throughout the experiment at a value of 10 μL min^–1^. A change of 10^4^ RU corresponding to 1 μg cm^–2^ of binding capacity was considered, based on the literature.^73^ Brush-coated SPR chips were mounted on plastic sensor chip supports, docked, and left to equilibrate with PBS until a stable baseline was obtained. A set of protein concentrations ranging from 1 to 10 μg mL^–1^ was prepared by serial dilutions, with the solvent matching the running buffer to reduce RI drifts. All injections were carried out incrementally, starting from the lowest concentration. Upon equilibration, 50 μL of protein solution was injected for 5 min, followed by a washing step with running buffer for 10 min. Prior to each subsequent injection, regeneration with 2 M NaCl and further equilibration were performed.

### Quartz Crystal Microbalance with Dissipation Monitoring

To analyze BMP binding to polymer brushes, PSPMA-functionalized piezoelectric SiO_2_ or Au-coated quartz crystals with a fundamental frequency (*f*_0_) of 5 MHz were employed. The linear relationship between the added hydrated mass layer (Δ*m*) and resonance oscillation frequency shift (Δ*f*) is described by the Sauerbrey equation
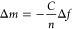
where *C* is the mass sensitivity constant, equal to 18 ng cm^–2^ Hz^1–^ for 5 MHz crystals. In addition, QCM-D can measure energy loss or dissipation, which refers to the decay of crystal oscillations when the power is turned off. The shift in dissipation (Δ*D*) provides insights into the structural and viscoelastic properties of the surface and characterized adsorbed layers.

Prior to use, the crystals were immersed in an EDTA solution (10 mM, pH 7.3) for 10 min. A QCM-D sensor system (Q-Sense Explorer, Biolin Scientific, Sweden) was used to monitor the adsorption of BMP2 (10 μg/mL) and BMP4 (10 μg/mL) in PBS onto the crystal and record the frequency (from third to 13th harmonics) and dissipation shifts. Each harmonic has a specific penetration depth, described by the following equation
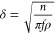
where δ is the penetration depth, f is the resonating frequency (related to the overtone number, i.e., *f*_3_ = *f*_0_ × 3), η is the viscosity, and ρ is the density of the film. PBS was applied as running buffer, and NaCl (1 M) was used to remove any residual BMP left on the brushes before the next injection. BMPs were injected rapidly (100 μL/min) for 90 s until a plateau was achieved with a peristaltic pump (IPC4, Ismatec), while buffer solutions were continuously injected (15 μL/min).

The data have been analyzed with Dfind software (Biolin Scientific) by using the Broadfit model, to fit the data and extract the mass adsorption values.

### Dermal Fibroblast Culture and Seeding

Human dermal fibroblast cells (HCA2; hTERT-immortalized human dermal fibroblast cell line^74^) were cultured from passage 22 in T75 flasks with Dulbecco’s modified Eagle’s medium (DMEM) supplemented with 10% FBS, 1% 2 mM l-glutamine, and 1% PS in an incubator (37 °C and 5% CO_2_). The medium was aspirated and replaced every 2–3 days. When 70–90% confluency was reached, fibroblasts were harvested with trypsin (0.25%) and Versene solutions (EDTA Na_4_, 0.2 g/L) in PBS in a ratio of 1:9, centrifuged, counted, and resuspended in DMEM in a T75 flask at the desired density. For cell seeding onto substrates, fully confluent fibroblast cells (HCA2) cultured at 37 °C at 5% CO_2_ were seeded at a density of 60,000 cells per well (24-well plates). Cells were left to adhere for 24 h in the incubator, and resulting cell cultures were examined via bright field microscopy on days 1, 3, 5, and 7. The culture medium was exchanged every other day using ascorbic acid (l-ascorbic acid-2 phosphate; 50 μg/mL)-supplemented DMEM.

### Cell Denudation and Immunostaining

After 10 days of culture, cell denudation was carried out by using an extraction buffer. The plates were slightly tilted to aspirate media using a sterile Pasteur pipet and were washed with 2 mL of PBS once. The extraction buffer was prepared with PBS (48.8 mL) containing 0.25% (v/v) Triton X-100 (250 μL) and 10 mM ammonium hydroxide (250 μL). This extraction buffer solution was prewarmed in a 37 °C water bath, and 1 mL of this solution was then carefully added to each well. The coverslips were gently lifted with a pipet tip and tweezers so that the buffer could reach under them. It was left for 4 min for cell lysis, as confirmed by bright field microscopy (Leica DMI8 epifluorescence microscope). Then, half of the buffer was carefully removed using a Pasteur pipet. Approximately 2 mL of PBS without Ca^2+^ and Mg^2+^ was added to each well. These steps were repeated until no intact cells were seen under the microscope. 10 mL of 10 μg/mL DNase I solution (Roche) was freshly prepared by adding 10 μL of DNase I stock solution (10 mg/mL) to 10 mL of sterile PBS. 2 mL portion of this DNase I solution was then added to each well to digest the DNA residues and incubated for 30 min at 37 °C. The denuded CDMs were washed with 2 mL of PBS twice.

The plates were then tilted slightly to aspirate the PBS carefully using a Pasteur pipet. 1 mL of 4% PFA was added to each well, to fix for 20 min at room temperature. It was then carefully pipetted away and washed with PBS twice. 4% (wt/vol) BSA blocking solution was freshly prepared by diluting 2 g of BSA in 50 mL of PBS. This solution was filter sterilized through a 0.45 μm filter before adding 2 mL to each well for 1 h at room temperature. By using tweezers and a pipet tip, each coverslip was gently removed from the wells. Excess solution was dried off and they were then placed in a humidified chamber. 100 μL of the primary antibodies in BSA solution, anticollagen-I, and antifibronectin in 1/1000 dilution was added to each coverslip, and the humidified chamber was left at 4 °C overnight. The next day, each coverslip was washed by dipping it in PBS (approximately 10 times), and the excess was dried off. Then, 100 μL of the secondary antibodies in BSA solution, Alexa Fluor 488 (goat antimouse antibody), and Alexa Fluor 555 (donkey antirabbit antibody) in 1/1000 dilution was added to each coverslip and was incubated for 1 h in the dark at room temperature. After this, each coverslip was washed again by dipping (approximately 10–15 times) in PBS first and then in deionized water. After carefully removing the excess solution, 2–3 coverslips per slide were mounted using 10 μL of Mowiol and were allowed to set overnight.

### Fluorescence Microscopy

Fluorescence microscopy images were acquired with a confocal microscope (ZEISS LSM710 confocal and Elyra PS.1 superresolution microscope using Zen 2012 sp5) at a magnification of 63× oil on a lens. The density of the matrix was determined by measuring the interfiber distance and the fluorescence intensity of the pixels on each substrate.

### Statistical Analysis

Statistical analysis was carried out using OriginPro 9, through one-way ANOVA with Tukey’s test for posthoc analysis. Significance was determined by *P < 0.05, **P < 0.01, ***P < 0.001, and n.s. (nonsignificant). A full summary of statistical analysis is provided in the Supporting Information.

## Results and Discussion

**Figure 1:**
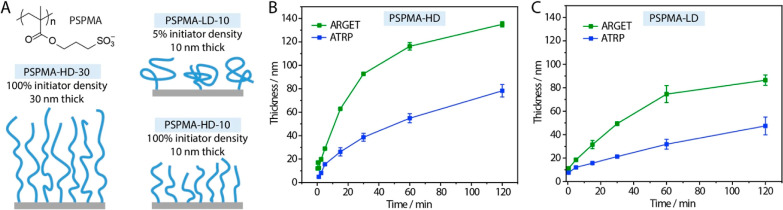
(A) Schematic representation of the PSPMA brushes studied, with different thicknesses (30 or 10 nm) and grafting densities (5 and 100% densities of initiator molecules). (B) Polymerization kinetics of high-density PSPMA brushes via ATRP (blue) and ARGET (green). ARGET conditions: 1:1H_2_O/EtOH 900:10:1:15:9.3 SPMA/bipyridine/CuBr_2_/ascorbic acid/KCl. ATRP conditions: 900:10:1:10 SPMA/bipyridine/CuBr_2_/CuCl. (C) Polymerization kinetics of low-density PSPMA brushes via ATRP (blue) and ARGET (green). Error bars are s.e.m; *n* = 3.

The synthesis of surface-initiated PSPMA brushes is typically carried out via ATRP in aqueous/methanol mixtures, allowing brushes with thicknesses >100 nm to be readily prepared.^69,70^ The kinetics of brush growth can be readily adjusted through the ratio of Cu(I)/Cu(II) complexes used and that of methanol/water. ARGET is an attractive alternative to conventional ATRP catalytic systems, as it, in principle, enables the toleration of some oxygen.^44,75^ Indeed, ARGET has been applied to the synthesis of a broad range of polymer brushes, including PMMA, polystyrene, PGMA, and POEGMA brushes, among others.^76−78^ As the kinetics of polymerization could be expected to vary significantly, compared to ATRP, the growth of PSPMA brushes generated via ATRP and ARGET was first compared (Figure 1).

In agreement with the literature, the growth of PSPMA brushes from dense and sparse monolayers of initiators (monolayers composed of 100 and 5% of silane ATRP initiators, respectively) was found to be relatively linear, with a steady increase in thicknesses to 60 and 40 nm over 120 min of polymerization, respectively (Figure 1B,C). Interestingly, the thickness of PSPMA brushes generated via ARGET, at both densities, was found to be significantly higher, although a clear reduction in brush growth was observed after 60 min. Although such thicknesses are in agreement with the growth of other hydrophilic brushes by ARGET, including POEGMA and PDEAEMA,^78,79^ such enhancement in the rate is not typically reported, although direct quantitative comparisons between a surface-initiated activator regenerated by electron transfer (SI-ARGET) and SI-ATRP have not been systematic. This enhanced kinetics was observed on brushes grown from both high- and low-density initiators, with comparable rate accelerations (just under double the thickness achieved by ARGET compared to ATRP). Comparable growth kinetics were observed for brushes generated from gold substrates coated with ATRP-thiol initiators (Figure S1), although with less reproducibility at later time points, perhaps resulting from slight variations in oxygen exposure when brushes were generated in separate batches (no precaution was taken to exclude oxygen during polymerization). Therefore, ARGET appears as an ideal polymerization system to carry out PSPMA brush growth from surfaces in deoxygenated solutions but without elimination of atmospheric oxygen (brush growth was carried out in multiwell plates in the case of ARGET, see Methods).

**Figure 2:**
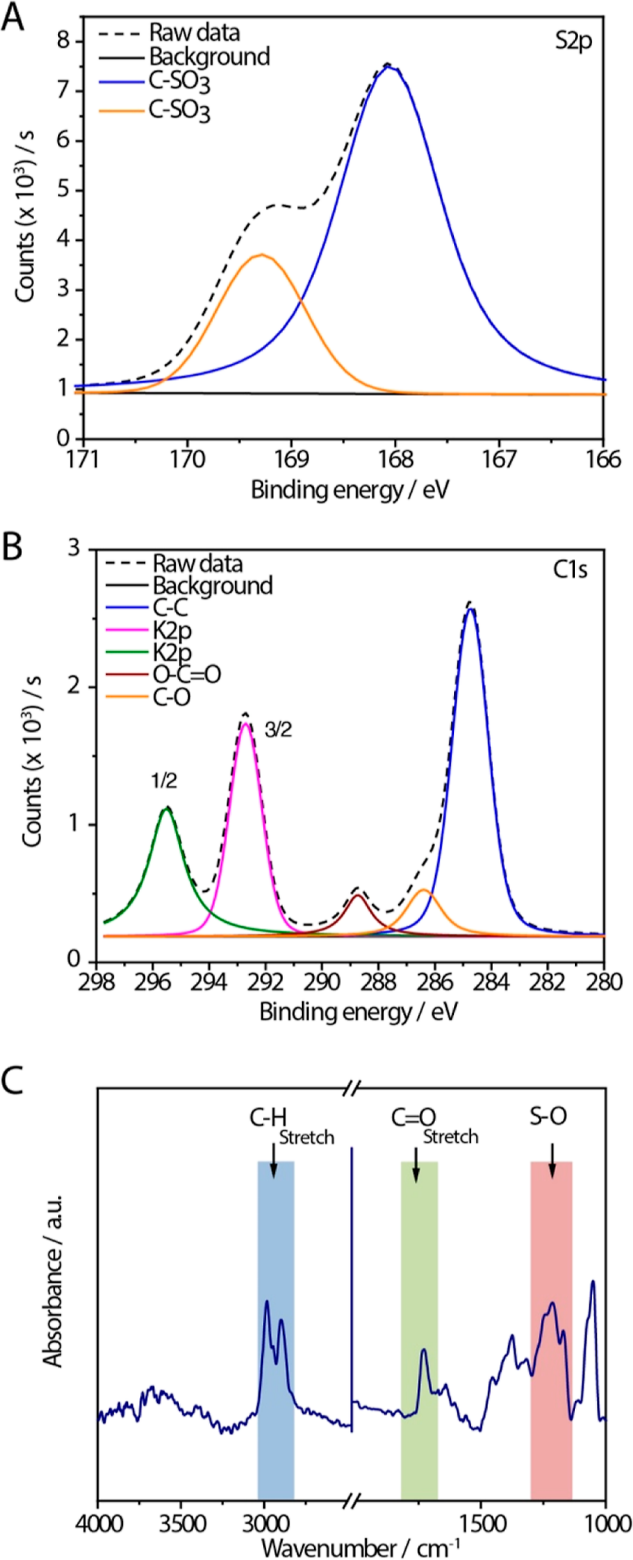
Chemical characterization of PSPMA brushes generated via ARGET-ATRP. Deconvoluted high-resolution XPS spectrum for (A) S 2p and (B) C 1s. C. Fourier transform infrared (FTIR) spectrum of ARGET-generated PSPMA brushes, with assignment of some of the vibrational bands.

To confirm the chemistry of the brushes generated, elemental analysis was carried out using X-ray photoelectron spectroscopy (XPS) (Figures 2A,B, S2, and S3). The wide scan survey of ARGET-generated PSPMA brushes features the presence of all elements expected from the chemical structure of this polymer with potassium counterions (Figure S2). Atom compositions measured were in excellent agreement with those calculated for PSPMA, with a minor discrepancy due to the presence of silicon, presumably owing to defects in the brush coating. The C 1s and K 2p peaks, partially overlapping in the 280–298 eV scans, are fully consistent with the expected chemistry of PSPMA brushes and S 2p peaks at 168 and 169.5 eV are confirming the presence of sulfonate residues at high densities in this coating (Figure 2A,B).^80^ These results are also in excellent agreement with the spectra obtained from PSPMA brushes generated via conventional ATRP (Figure S3), confirming the achievement of brushes with comparable chemical structures via both polymerization techniques.

However, ATRP-generated brushes also displayed Cu and N components (e.g., Cu 2p and N 1s peaks) that are proposed to result from residual catalysts and associated ligands (bipyridine; Figure S4). Upon incubation in buffer (PBS), these components are displaced, irrespective of the brush thicknesses studied, indicating that simple exchange with electrolytes is sufficient to remove traces of catalysts present and recover a pristine PSPMA brush chemistry, although replacing potassium salts with sodium counterions. Interestingly, sparse brushes did not feature residual catalysts, perhaps suggesting that the high density of PSPMA brushes at full density of ATRP initiators constitutes an environment-stabilizing molecular catalytic species.

Grazing angle FTIR spectroscopy further confirmed the chemistry of the brushes generated (Figures 2C and S5). Bands typically associated with PSPMA brushes, including those corresponding to asymmetric sulfonate, stretch near 1200 cm^–1^ and symmetric sulfonate stretching at 1045 cm^–1^ can be clearly seen in ARGET-initiated as well as ATRP-initiated brushes, in addition to bands more broadly associated with methacrylate polymer brushes (C–H stretching bands near 2900 cm^–1^, carbonyl stretching bands at 1730 cm^–1^, CH_2_ bending vibrations near 1450–1500 and 740 cm^–1^, and C–O stretching of esters at 1245 cm^–1^^70^). Comparable spectra and vibrational features were observed for both ARGET- and ATRP-initiated brushes but with distinct patterns, potentially reflecting the hydration state of the corresponding brushes, or their association with residual catalysts (ATRP-initiated brushes had not been cleared from complexes prior to FTIR characterization). Overall, our data confirm the chemistry of PSPMA brushes generated via ARGET, positioning this synthetic approach as particularly attractive for the coating of a wide range of interfaces with this polymer, in scalable formats.

**Figure 3:**
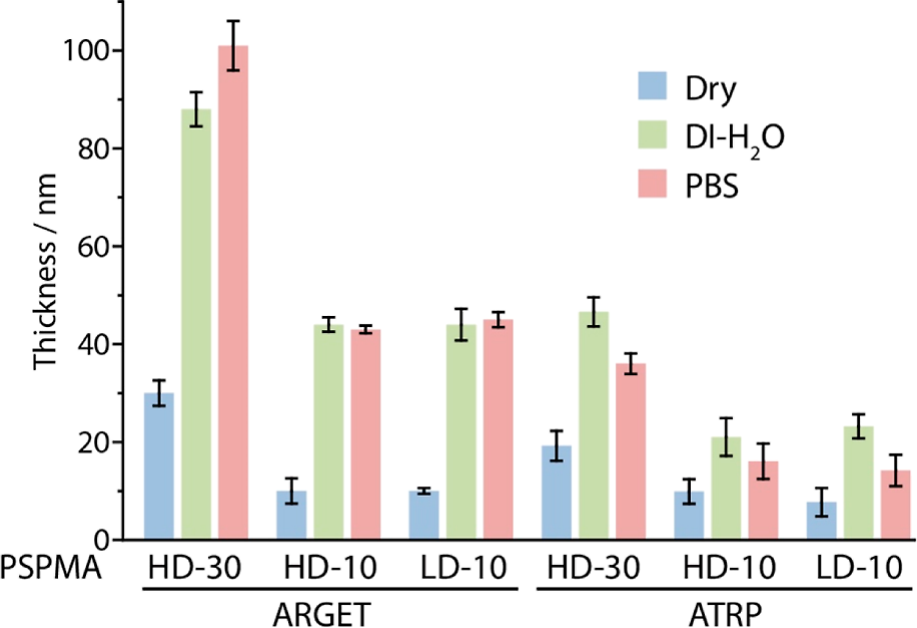
Swelling behavior of high- and low-density PSPMA brushes. Comparison between swelling of brushes generated from silicon substrates, in different conditions, via ARGET and ATRP.

The swelling of PSPMA brushes generated via ARGET and ATRP was examined next via ellipsometry (Figure 3). Upon immersion in deionized water, PSPMA brushes swell considerably, from 30 to 88 nm and from 20 to 42 nm for ARGET- and ATRP-initiated brushes, respectively. Upon exposure to PBS, swelling increased slightly in the case of ARGET-initiated brushes and decreased slightly in the case of ATRP-initiated brushes. In addition, swelling was more pronounced in the case of thin (HD-10) and sparse (LD-10) brushes, particularly in ARGET-initiated systems. These swellings, weaker than those reported for cationic polyelectrolyte brushes,^72,79^ are consistent with those reported for the literature for ATRP-initiated PSPMA brushes.^81^ The enhanced swelling of PSPMA brushes generated via ARGET, particularly those with lower thicknesses and sparser densities, implies a reduced initiation density and/or increased polydispersity, as predicted by self-consistent field theoretical studies.^82−84^ Although this could not be tested in our study, owing to the limited material that can be recovered from planar substrates and the difficulty of characterizing the molecular weight of strong polyanionic materials by size exclusion chromatography, these results imply that brushes grown via an ARGET mechanism display longer chains (enhanced rates of polymerization) but also higher polydispersity and lower surface density compared to those generated via an ATRP mechanism. This may result from the precise regulation of the ratios of Cu(I)/Cu(II) species in ARGET, compared to ATRP, and the impact that this has on initiation rates, the persistence of radicals, and recombination events.

**Figure 4:**
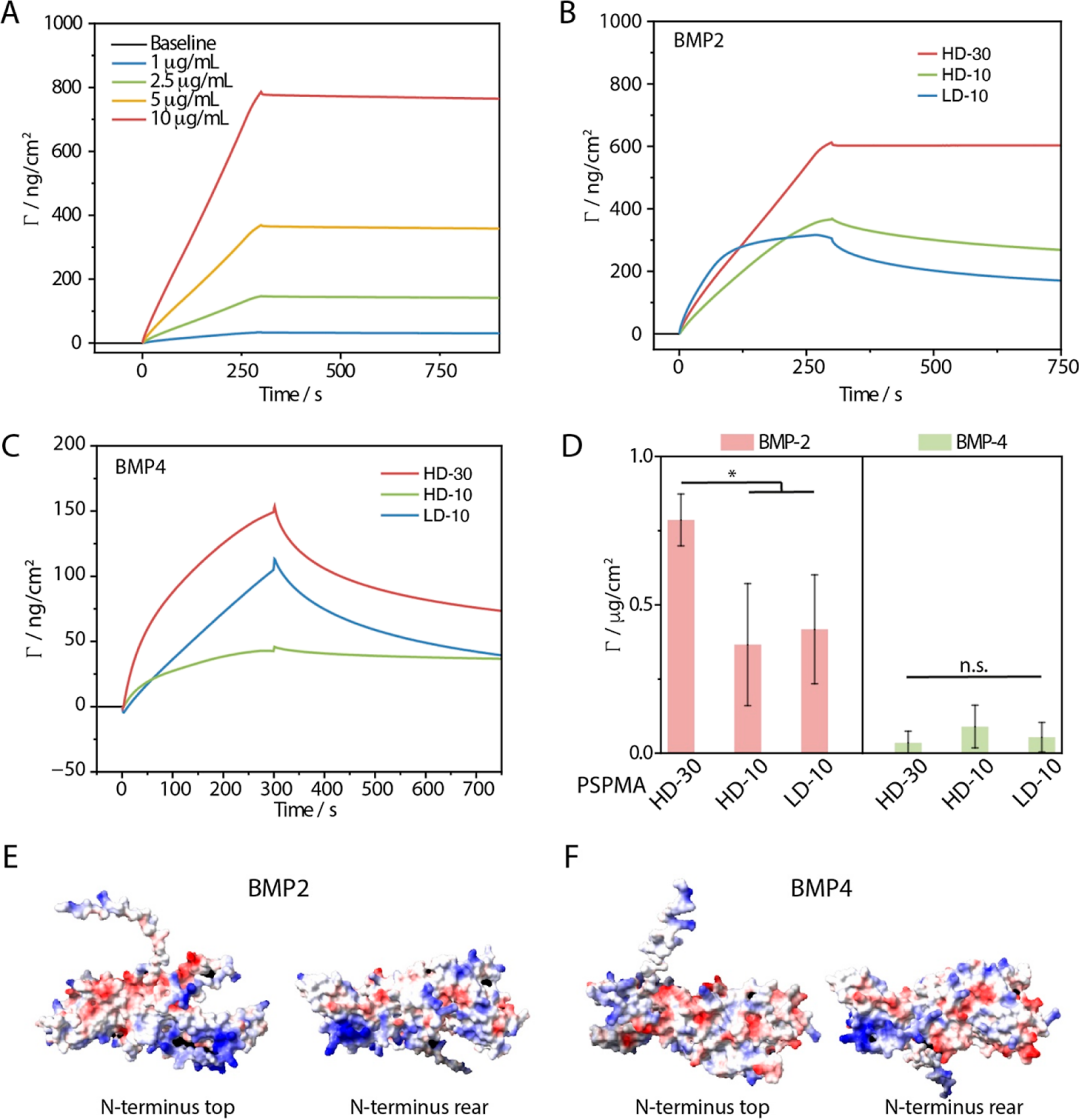
SPR traces obtained for the adsorption of BMP2 at the surface of PSPMA brushes generated via ARGET. (A) SPR traces showing the absorption of BMP2 on dense 30 nm brushes (HD-30) at different protein concentrations. (B) Traces showing the adsorption of BMP-2 on sparse and dense brushes and (C) that of BMP4 on sparse and dense brushes. (D) Corresponding protein adsorption (μg/cm^2^) measured after washing of the substrates (at 10 μg/mL protein concentrations). (E,F) AlphaFold structures (obtained from UniProt) of BMP2 and BMP4 (respectively), displaying the electrostatic surface potential, with the N-terminus of the protein pointing toward the top or the rear of the plane of the image and indicating more prominent positively charged patches associated with BMP2.

Having explored some of the physicochemical properties of PSPMA brushes generated via ARGET and ATRP mechanisms, their ability to bind and sequester BMPs was investigated next, first via SPR (Figures 4 and S6–S8). BMP2 rapidly adsorbed at the surface of dense and thick PSPMA brushes (HD-30), giving rise to a stable anchorage of proteins at surface densities (Γ) of 756 ± 64 ng/cm^2^ (upon injection at 10 μg/mL), 5 min after injection. This density corresponds to 0.1 molecule per nm^2^, indicating the formation of relatively dense monolayers. Thinner brushes (10 nm), at either high or low densities (HD-10 and LD-10, respectively), resulted in approximately half of the adsorption levels measured for HD-30 (370 ± 176 and 268 ± 57 ng/cm^2^, respectively). However, the rates of adsorption to sparser brushes (LD-10) were found to increase, compared to those of dense brushes of comparable dry thicknesses (HD-10). Considering the comparable swelling of both brushes, the reduction in surface adsorption compared to HD-30 brushes indicates some level of infiltration of BMP2 within thick PSPMA brushes but a more accessible binding to sparser brushes, presumably able to accommodate some level of infiltration and conformational rearrangement. Presumably, this could also account for the partial desorption observed for both HD-10 and LD-10 PSPMA brushes. Overall, this adsorption behavior is reminiscent of the adsorption of oligonucleotides of varying sizes, adsorbing to cationic polyelectrolyte brushes in a size- and density-dependent manner.^53^

In comparison, BMP4 adsorption was far more limited, even to thick dense brushes (HD-30), with maximum surface adsorption densities in the range 37–92 ng/cm^2^ (Figures 4C,D and S7). In addition, protein adsorption was found to be weaker, with significantly higher off rates, corresponding to molecular desorption, upon washing with buffer. Together, these observations indicate significant differences in the adsorption levels and associated equilibrium drive of BMP2 and BMP4 to PSPMA brushes. Considering the relatively high homology of both proteins and similarity of their architecture, this significant difference in adsorption is surprising; however, analysis of the surface electrostatic densities of both proteins (generated using ChimeraX, based on the AlphaFold-generated structure obtained from UniProt) revealed the occurrence of charged patches in BMP2 that are absent from the surface of BMP4 (Figure 4E,F). Charged patches were proposed to be responsible for the adsorption of proteins to polyelectrolyte brushes, even in the case of like-charged protein and polymer couples (e.g., in the case of albumin, lysozyme, β-lactoglobulin, and RNase^56,67,85,86^). Therefore, we propose that positively charged patches are responsible for the strong and stable adsorption of BMP2 onto PSPMA brushes, in contrast to the weaker and less stable adsorption of BMP4.

PSPMA brushes generated via ATRP resulted in comparable profiles of adsorption, with BMP2 adsorbing at significantly higher densities on dense brushes, compared to BMP4 (Figure S8). However, while BMP4 adsorption was comparable on all three types of brushes studied (HD-30, HD-10, and LD-10), as in the case of ARGET-generated brushes, BMP2 adsorption to high density thick and thin brushes (HD-30 and HD-10) was comparable. This could suggest that the reduced swelling of ATRP-generated PSPMA brushes may limit infiltration and, in turn, impact the ultimate adsorption levels of BMP2 to corresponding brushes. This is consistent with the observation that BMP2 adsorbed to reduced levels overall on ATRP-generated PSPMA brushes, which displayed reduced swelling compared to that of ARGET-generated brushes of comparable densities and thickness.

**Figure 5:**
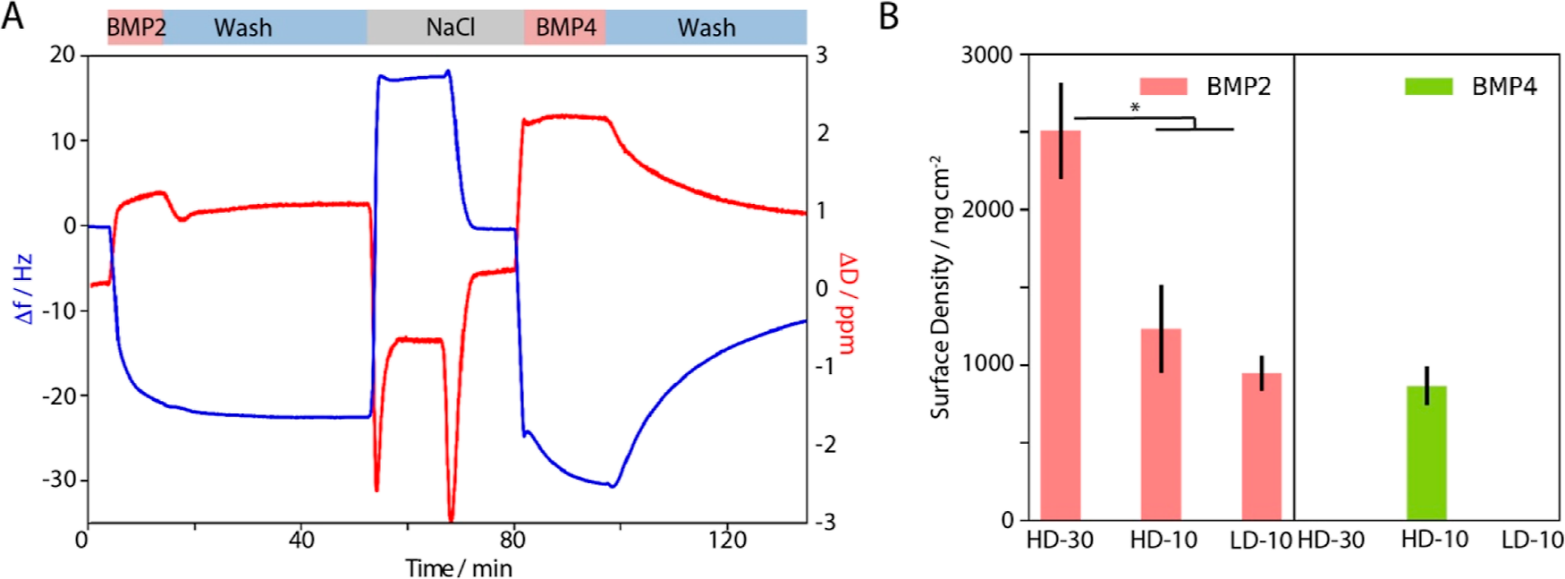
(A) QCM-D traces are used to monitor the binding of BMP2 and BMP4 on dense thin PSPMA brushes (HD-10) on gold-coated crystals. BMPs were injected at 10 μg/mL. Blue lines correspond to frequency shifts and red lines correspond to dissipation shifts of the third overtone. (B) Quantification of the adsorbed masses of BMP2 and BMP4 on all types of brushes on the SiO_2_ crystal, using the Dfind viscoelastic model. As BMP4 did not show any significant frequency shift on the thick and sparse brushes, the software was not able to fit the QCM-D data. Results are averages with standard deviations (*N* = 3; * *P* < 0.05).

**Figure 6:**
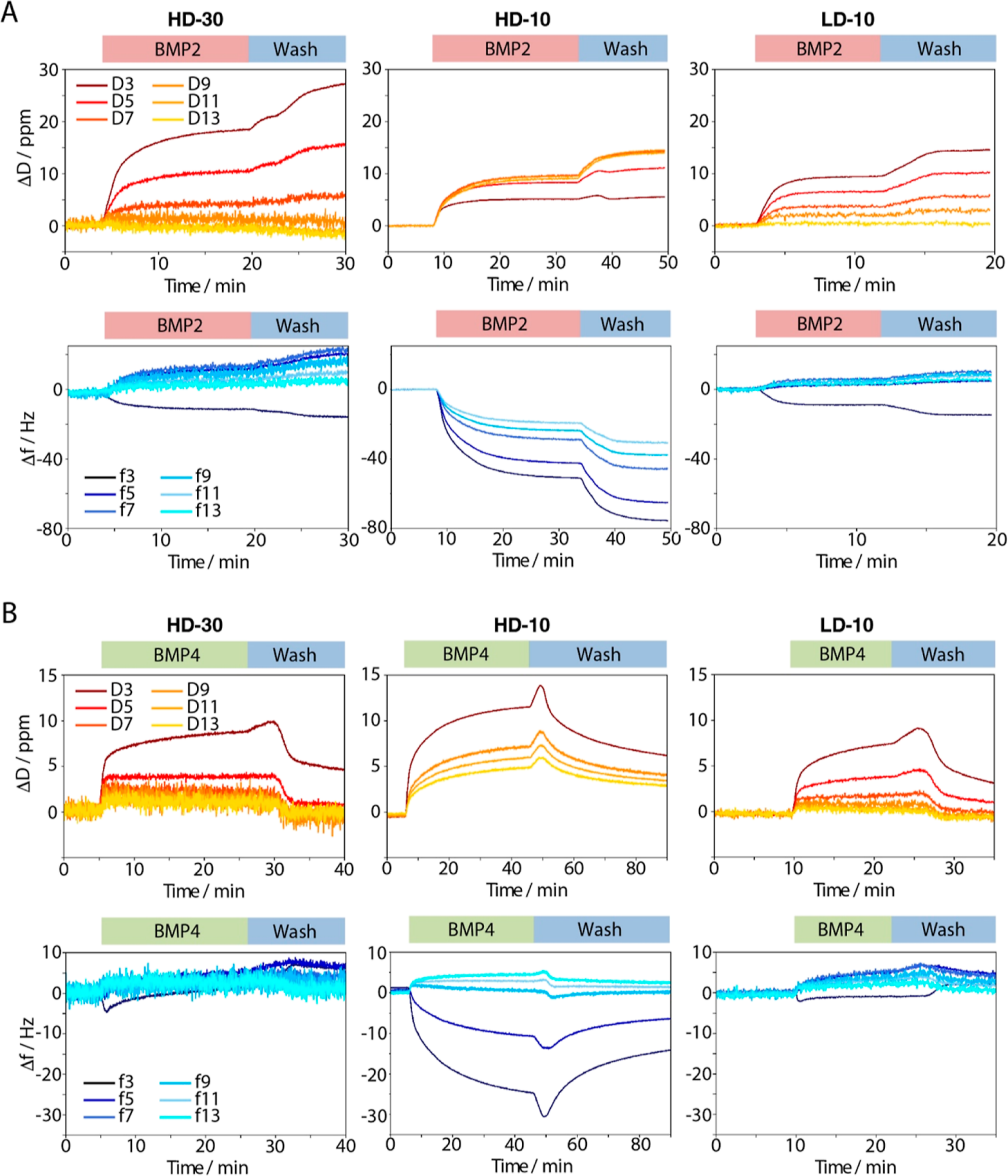
QCM-D study of BMP-2/-4 binding to PSPMA brushes with different lengths and densities, generated from the SiO_2_ crystal. QCM-D traces of representative adsorptions of BMP2 (A) and BMP4 (B) binding to thick, thin, and sparse PSPMA brushes (HD-30, HD-10, and LD-10). Dissipation shifts (Δ*D*) are presented in red and frequency shifts (Δ*f*) are presented in blue. BMPs were injected at 10 μg/mL, at rates of 100 μL/min during 90 s before stabilization. The shifts observed during rinsing are due to a residual BMP inside the entry tubings of the QCM-D.

To explore further BMP adsorption onto PSPMA brushes, we used QCM-D (Figures 5 and 6, and S9 and S10). Upon binding of both BMPs to dense PSPMA brushes (HD-10), a marked reduction in frequency was observed together with a significant increase in dissipation (Figure 5A). This indicates an increase in viscoelasticity of the brush–BMP complex, compared to that of the pristine brush, particularly in the case of BMP4, which could be due to the binding of the BMPs at the top of the brush. Upon washing of the brush–BMP complex with buffer, a substantial recovery of the frequency and dissipation was observed in the case of BMP4, in agreement with the weaker binding and surface densities measured by SPR. Overall, surface densities extracted from QCM-D experiments for both BMP2 and BMP4 are aligned with SPR observations, indicating significantly higher adsorption levels with dense and thick HD-30 brushes and significantly higher binding of BMP2 (Figure 5B). The higher surface densities quantified by QCM-D reflect the sensitivity of this technique to hydrated mass, although it is not clear whether this originates from the hydrated sphere of the proteins adsorbed alone, or whether this is also associated with conformational changes and swelling of the brushes too, as was evidenced during the binding of oligonucleotides to PDMAEMA brushes, via neutron reflectometry.^87^

A more detailed analysis of the QCM-D data revealed further insights into the adsorption process of BMPs to PSPMA brushes and the impact of their architecture. On thick dense brushes (HD-30), upon binding of BMP2, the third harmonic frequency decreased, but other harmonics increased, and the dissipation signal decreased with increasing harmonic numbers (Figure 6A). This may be explained by the binding of BMP2 only at the top of the brush coupled with some release of solvent from the bulk of the brush. In contrast, with thin dense brushes (HD-10), the smaller the overtone, the smaller the dissipation shift. This may be explained by an improved penetration of BMP2 inside the brush (or nonspecifically at the surface of the crystal^88^). This observation is in agreement with the reduced kinetics of adsorption observed by SPR (Figure 4B).

In agreement with the SPR data, binding of BMP4 to PSPMA brushes was found to be significantly weaker than the binding of BMP2 (Figure 6B). Upon BMP4 binding, changes in dissipation and frequency shift were less pronounced than those detected for BMP2, on all three brush types, although trends (e.g., comparing HD-30, HD-10, and LD-10) were qualitatively similar to those described above for BMP2. However, one major difference clearly observed was that, upon washing, dissipation and frequency shifts did not stabilize, as in the case of BMP2, but reduced. This implies a weaker binding of BMP4, with significant desorption during washing steps.

QCM-D also enabled probing of the impact of the chemistry of the underlying substrate on BMP binding (Figure S9). In contrast to trends observed for silica substrates, it was observed that BMP4 and BMP2 (in particular) bound more strongly to sparse brushes (LD-10) generated from gold substrates, compared to those generated from silica substrates. Frequency shifts were more pronounced for these brushes, with reduced dissipation components, indicating an increased binding and suggesting the formation of a more rigid protein–brush complex.

**Figure 7:**
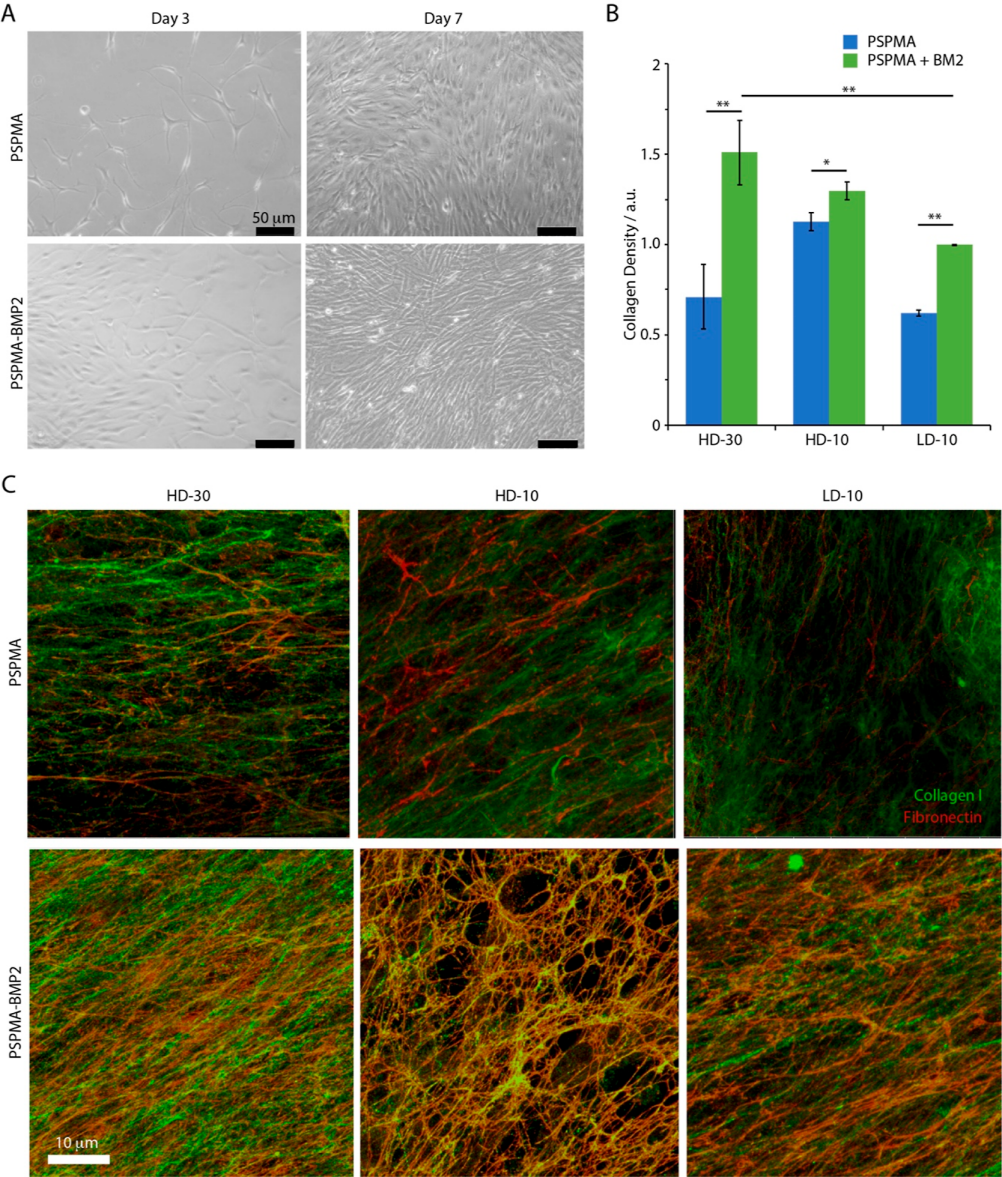
(A) Dermal fibroblasts cultured at the surface of dense thick PSPMA brushes (HD-30), with and without BMP2 coatings (10 μg/mL), at days 3 and 7 (bright field microscopy images). (B) Density of collagen I matrix deposition quantified from fluorescence microscopy images (immunostained samples), after 7 days of culture of dermal fibroblasts at the surface of PSPMA brushes with and without adsorbed BMP2. (C) Corresponding confocal microscopy images of fibronectin and collagen I fiber mats.

Finally, the impact of BMP adsorption on cell phenotypes was explored. To do so, we focused on BMP2-functionalized PSPMA brushes, owing to its more extensive adsorption and stability, and examined the impact of the brush architecture on the cell response to growth factor binding. BMPs play an important role in skin development and regulate some of the processes orchestrating interactions between dermal and epidermal compartments,^89^ as well as controlling matrix remodelling in other contexts.^90,91^ The cytotoxicity of patterned PSPMA brushes had previously been investigated, in primary keratinocytes, and was found to be negligible, with comparable viabilities to plastic controls.^81^ Hence, we examined the impact of BMP2 immobilization on PSPMA brushes on the deposition of ECM by dermal fibroblasts. Fibroblasts seeded on PSPMA brushes in the absence of BMP2 spread very slowly and sparsely, regardless of the thickness and density of the brush (Figures 7A, S11, and S12). This was particularly striking on dense and thick polymer brushes (HD-30), for which few spread cells could be seen even at day 3 postseeding. However, by day 7, all substrates were fully covered by dense fibroblast monolayers. We noted the formation of cell clusters on thin PSPMA brushes, whether they were generated from dense or sparse initiator monolayers. In contrast, fibroblasts spread more rapidly on all of the PSPMA brushes coated with BMP2. Regardless of their architecture and the adsorption of BMP2, all substrates were found to be coated by dense fibroblast layers after 7 days of culture. We note that, the morphology of the PSPMA brushes was found to be smooth and homogeneous prior and after BMP2 adsorption (Figure S13). Similarly, we noted only modest changes in the roughness of corresponding surfaces, as probed by AFM (Figure S14; roughnesses of 0.22 ± 0.04 and 0.33 ± 0.06 nm were measured for PSPMA and PSPMA-BMP2, respectively). Therefore, changes in nanostructures are unlikely to contribute significantly to the modulation of cell adhesion to BMP2-functionalized PSPMA brushes, as is reported at a broad range of nanotextured biointerfaces.^92^

The deposition of the extracellular matrix was next examined (Figure 7B,C and Figure S15). The density of collagen I assembled at the surface of PSPMA brushes was found to be impacted by not only the architecture of the brush but also the presence of BMP2 (Figure 7B). Hence, collagen I deposition by dermal fibroblasts was found to be more extensive on HD-30 PSPMA brushes coated with BMP2, compared to sparser brushes or uncoated brushes. This implies that brushes displaying higher densities of BMP2 promote collagen matrix deposition. Furthermore, the densely negatively charged surface of PSPMA brushes is intrinsically conducive to the adsorption of basic collagen I molecules (with an IP of 8–9^93,94^) and this protein was previously found to adsorb strongly at the surface of PSPMA brushes.^81^ In addition to such direct mechanisms, BMP2 is known to bind ECM proteins such as fibronectin.^95^ Fibronectin fiber networks are in turn considered to be preliminary to the assembly of more complex ECM fibrous networks and were reported to precede the assembly of collagen I fibers.^96,97^

Examination of ECM fibers deposited at the surface of polymer brushes confirmed the abundance of rich and dense fibronectin fiber networks (Figures 7C and S15). Unlike collagen I, fibronectin was not previously found to adsorb to PSPMA brushes,^81^ owing to its relatively low IP (near 6^98^). In agreement with this lack of direct interaction, relatively sparse fibronectin matrices were observed at the surface of all types of PSPMA brushes, with fibronectin density overall remaining low and relatively high gaps between fibronectin fibers (Figure S16). However, fibronectin adsorption was found be significantly enhanced by the presence of BMP2 at the surface of PSPMA brushes, resulting in denser fiber mats (Figure S16). In turn, the collagen fibers assembled at the surface of BMP2-coated PSPMA brushes, particularly with high density and thickness, were both more abundant and more tightly assembled. Therefore, these observations suggest that the combination of strong negative electrostatic potential and ability to capture BMP2 enables the assembly of dense and tightly packed ECM fibers.

## Conclusions

The growth of PSPMA brushes via ARGET was found to be well controlled, even in the absence of inert atmosphere (but with prior degassing of the polymerization solution), therefore greatly simplifying the protocols used for the controlled polymerization of this type of brushes. Such conditions should be translatable to a broad range of contexts, beyond the multiwell plate format applied in the current study. Interestingly, the kinetics of PSPMA brush growth via ARGET was found to be enhanced compared to ATRP, in otherwise comparable conditions, perhaps reflecting a lack of control of the precise ratio of Cu(I) to Cu(II) species and associated rates of polymerization. In turn, the swelling behavior of PSPMA brushes was not only found to depend on the density and thickness but also the catalytic system used. This could indicate that the faster kinetics of brush growth resulting from ARGET leads to more polydisperse, and perhaps sparser, brushes with increased swelling. However, the chemistry of PSPMA brushes generated via both methods (SI-ARGET and SI-ATRP) was found to be comparable, as confirmed by XPS and FTIR.

In turn, the architecture of the brushes generated and their solution morphology regulated the adsorption of BMP2. Dense and thick PSPMA brushes led to significant levels of adsorption of BMP2, while this was reduced on thinner brushes, regardless of their density. In contrast, BMP4 did not adsorb as significantly, perhaps reflecting differences in the charge distribution and associated electrostatic potential at the surface of the corresponding proteins. QCM-D data also indicated the surface adsorption of BMP2 to thick dense brushes, with some evidence of infiltration within thinner coatings, perhaps as a result of increased polydispersity at early polymerization time points. Overall, these results indicate a predominantly secondary surface adsorption to polymer brushes, particularly with high densities and thicknesses. This is in contrast with the adsorption of nucleic acid molecules adsorbing deeply into cationic brushes.^87^

BMP2 and BMP4 interact with GAGs through their N-terminal domains. Both exhibit typical Cardin–Weintraub sequences in their N-terminal region, with [XBBXBX] and [XBBBXXBX] motifs, respectively (B: basic residue; X: noncharged residue).^99^ Ruppert et al. demonstrated that a BMP2 variant, where the N-terminal residues 1–12 have been substituted by a dummy sequence, exhibited a negligible interaction with Hep.^100^ Although the Hep (and heparan sulfate)-binding site of BMP2 has been well characterized, our recent findings indicate that it exhibits certain degrees of flexibility in binding to various sulfation patterns of heparan sulfate.^101^ In light of these observations, we hypothesized that strongly negatively charged polymeric brushes presenting sulfonate residues may also facilitate BMP2 binding. We indeed propose that a similar mechanism regulates the adsorption of BMP2 to PSPMA brushes. However, a detailed domain function analysis, for example, using the N-terminal truncated BMP2, is outside of the scope of this manuscript.

The differential binding of BMP2 and BMP4 deserves further discussion. Both proteins present the Hep/HS binding site at their N-terminal end, while other BMPs such as BMP5, 6, and 7 present the Hep binding site at their C-terminal end.^12^ In addition, the N-terminal sequences of BMP2 and BMP4 were found to be aligned. It was underlined that the Cardin–Weintraub sequences of BMP2 and BMP4 are similar but not identical. In particular, the sequence of the heparin-binding domain in BMP2 is QAKHKQRKRLKSSC, which differs from the sequence of BMP4 SPKHHSQRARKKNKNC. In the same study, peptides presenting the N-terminal sequences of both BMP2 and BMP4 were investigated. The peptides exhibited comparable binding affinities for HS. However, as illustrated in Figure 3B of this paper, the full BMP2 and BMP4 proteins exhibit disparate binding kinetics: BMP4 bound with slower kinetics, with respect to BMP2. Regrettably, the data in the paper have not been subjected to further analysis, as the authors concluded that the binding affinity was comparable. However, we hypothesize that the differential binding between these two proteins does not stem from the distinct N-terminal sequence. In this regard, the AlphaFold analysis illustrated in Figure 4E,F of our manuscript shows the electrostatic surface potential of both proteins. We noticed more positively charged regions in BMP2 with respect to BMP4. These differences may be responsible for the disparate binding affinities observed for HS and polymeric brushes.

The surface adsorption of BMP2 presumably leaves receptor-binding sites available for ligation.

As a result, BMP2 adsorption was found to impact the deposition of the matrix by fibroblasts adhering to the corresponding interfaces. The architecture of PSPMA brushes and the density of BMP2 captured at their surface had a significant impact on the assembly of both fibronectin and collagen I fibers. Although this latter molecule can adsorb onto PSPMA via electrostatic interactions,^81^ it is known to typically follow the preliminary adsorption of fibronectin in vitro.^96^ Therefore, it could be proposed that high BMP2 surface adsorption results in the capture of fibronectin and its deposition at the surface of PSPMA brushes, which in turn enhances further remodeling and deposition of collagen I, and potentially other proteins. However, whether this results from a direct ligation mechanism (through the binding of fibronectin to BMP2), or whether this arises through an upregulation of fibronectin secretion and assembly upon BMP2 ligation of the corresponding receptors, remains unknown.

Overall, this study demonstrates the excellent capacity of PSPMA brushes to capture heparin-binding growth factors, such as BMP2. The simplicity with which PSPMA can be generated via ARGET, even under ambient atmospheric conditions, without particular precautions to preclude oxygen contamination, will enable the coating of a broad range of materials and potentially implants with complex 3D shapes. Considering the importance of promoting matrix deposition in order to ensure soft tissue bonding to the surface of hard materials and implants, heparin-biomimetic PSPMA brushes may find application as coatings promoting tissue integration. Future studies could examine the adhesion of osteoblasts, and potentially mesenchymal stromal cells, at the surface of BMP-coated implants and the impact of such process on matrix remodelling and osseointegration.

## Supporting Information Available

Additional ellipsometry, XPS, FTIR, SPR, QCM, scanning electron microscopy, AFM, and microscopy data (PDF)

## References

[ref1] Fitzgerald K. A.; O’Neill L. A. J.; Gearing A. J. H.; Callard R. E. BMPs. In Cytokine FactsBook and Webfacts, 2001; p 168. 10.1016/B978-012155142-1/50034-8.

[ref2] Kobayashi T.; Lyons K. M.; McMahon A. P.; Kronenberg H. M. BMP Signaling Stimulates Cellular Differentiation at Multiple Steps during Cartilage Development. Proc. Natl. Acad. Sci. U.S.A. 2005, 102 (50), 18023–18027. 10.1073/pnas.0503617102. 16322106 PMC1312369

[ref3] Urist M. R.; Iwata H.; Ceccotti P. L.; Dorfman R. L.; Boyd S. D.; McDowell R. M.; Chien C. Bone Morphogenesis in Implants of Insoluble Bone Gelatin. Proc. Natl. Acad. Sci. U.S.A. 1973, 70 (12), 3511–3515. 10.1073/pnas.70.12.3511. 4357876 PMC427270

[ref4] Urist M. R. Bone: Formation by Autoinduction. Science 1965, 150 (3698), 893–899. 10.1126/science.150.3698.893. 5319761

[ref5] Reddi A. H. BMPs: From Bone Morphogenetic Proteins to Body Morphogenetic Proteins. Cytokine Growth Factor Rev. 2005, 16, 249–250. 10.1016/j.cytogfr.2005.04.003. 15949967

[ref6] Carreira A. C.; Alves G. G.; Zambuzzi W. F.; Sogayar M. C.; Granjeiro J. M. Bone Morphogenetic Proteins: Structure, Biological Function and Therapeutic Applications. Arch. Biochem. Biophys. 2014, 561, 64–73. 10.1016/j.abb.2014.07.011. 25043976

[ref7] Díaz P. U.; Hein G. J.; Belotti E. M.; Rodríguez F. M.; Rey F.; Amweg A. N.; Matiller V.; Baravalle M. E.; Ortega H. H.; Salvetti N. R. BMP2, 4 and 6 and BMPR1B Are Altered from Early Stages of Bovine Cystic Ovarian Disease Development. Reproduction 2016, 152 (4), 333–350. 10.1530/REP-15-0315. 27486268

[ref8] Guyot B.; Lefort S.; Voeltzel T.; Pécheur E. I.; Maguer-Satta V. Altered BMP2/4 Signaling in Stem Cells and Their Niche: Different Cancers but Similar Mechanisms, the Example of Myeloid Leukemia and Breast Cancer. Front. Cell Dev. Biol. 2022, 9, 787989 10.3389/fcell.2021.787989. 35047500 PMC8762220

[ref9] Hildebrand L.; Stange K.; Deichsel A.; Gossen M.; Seemann P. The Fibrodysplasia Ossificans Progressiva (FOP) Mutation p.R206H in ACVR1 Confers an Altered Ligand Response. Cell. Signalling 2017, 29, 23–30. 10.1016/j.cellsig.2016.10.001. 27713089

[ref10] Khodr V.; Machillot P.; Migliorini E.; Reiser J.-B.; Picart C. High-Throughput Measurements of Bone Morphogenetic Protein/Bone Morphogenetic Protein Receptor Interactions Using Biolayer Interferometry. Biointerphases 2021, 16 (3), 031001 10.1116/6.0000926. 34241280 PMC7614001

[ref11] Sefkow-Werner J.; Le Pennec J.; Machillot P.; Ndayishimiye B.; Castro-Ramirez E.; Lopes J.; Licitra C.; Wang I.; Delon A.; Picart C.; Migliorini E. Automated Fabrication of Streptavidin-Based Self-Assembled Materials for High-Content Analysis of Cellular Response to Growth Factors. ACS Appl. Mater. Interfaces 2022, 14 (29), 34113–34125. 10.1021/acsami.2c08272. PMC7614070 35849638

[ref12] Billings P. C.; Yang E.; Mundy C.; Pacifici M. Domains with Highest Heparan Sulfate-Binding Affinity Reside at Opposite Ends in BMP2/4 versus BMP5/6/7:Implications for Function. J. Biol. Chem. 2018, 293 (37), 14371–14383. 10.1074/jbc.RA118.003191. 30082319 PMC6139562

[ref13] Zhu Y.; Oganesian A.; Keene D. R.; Sandell L. J. Type IIA Procollagen Containing the Cysteine-rich Amino Propeptide Is Deposited in the Extracellular Matrix of Prechondrogenic Tissue and Binds to TGF-β1 and BMP-2. J. Cell Biol. 1999, 144 (5), 1069–1080. 10.1083/jcb.144.5.1069. 10085302 PMC2148200

[ref14] Ashe H. L. Type IV Collagens and Dpp Positive and Negative Regulators of Signaling. Fly 2008, 2 (6), 313–315. 10.4161/fly.7463. 19077532

[ref15] Chen R.; Wang J.; Liu C. Biomaterials Act as Enhancers of Growth Factors in Bone Regeneration. Adv. Funct. Mater. 2016, 26 (48), 8810–8823. 10.1002/adfm.201603197.

[ref16] Fu R.; Selph S.; McDonagh M.; Peterson K.; Tiwari A.; Chou R.; Helfand M. Effectiveness and Harms of Recombinant Human Bone Morphogenetic Protein-2 in Spine Fusion: A Systematic Review and Meta-Analysis. Ann. Intern. Med. 2013, 158, 890–902. 10.7326/0003-4819-158-12-201306180-00006. 23778906

[ref17] Migliorini E.; Weidenhaupt M.; Picart C. Practical Guide to Characterize Biomolecule Adsorption on Solid Surfaces (Review). Biointerphases 2018, 13 (6), 06D303 10.1116/1.5045122. 30352514

[ref18] Crouzier T.; Ren K.; Nicolas C.; Roy C.; Picart C. Layer-by-Layer Films as a Biomimetic Reservoir for RhBMP-2 Delivery: Controlled Differentiation of Myoblasts to Osteoblasts. Small 2009, 5 (5), 598–608. 10.1002/smll.200800804. 19219837

[ref19] Bouyer M.; Guillot R.; Lavaud J.; Plettinx C.; Olivier C.; Curry V.; Boutonnat J.; Coll J. L.; Peyrin F.; Josserand V.; Bettega G.; Picart C. Surface Delivery of Tunable Doses of BMP-2 from an Adaptable Polymeric Scaffold Induces Volumetric Bone Regeneration. Biomaterials 2016, 104, 168–181. 10.1016/j.biomaterials.2016.06.001. 27454063 PMC5937675

[ref20] Liu X. Q.; Fourel L.; Dalonneau F.; Sadir R.; Leal S.; Lortat-Jacob H.; Weidenhaupt M.; Albiges-Rizo C.; Picart C. Biomaterial-Enabled Delivery of SDF-1α at the Ventral Side of Breast Cancer Cells Reveals a Crosstalk between Cell Receptors to Promote the Invasive Phenotype. Biomaterials 2017, 127, 61–74. 10.1016/j.biomaterials.2017.02.035. 28279922 PMC5777630

[ref21] Kato K.; Sato H.; Iwata H. Immobilization of Histidine-Tagged Recombinant Proteins onto Micropatterned Surfaces for Cell-Based Functional Assays. Langmuir 2005, 21 (16), 7071–7075. 10.1021/la050893e. 16042424

[ref22] Sales A.; Khodr V.; Machillot P.; Chaar L.; Fourel L.; Guevara-Garcia A.; Migliorini E.; Albigès-Rizo C.; Picart C. Differential Bioactivity of Four BMP-Family Members as Function of Biomaterial Stiffness. Biomaterials 2022, 281, 121363 10.1016/j.biomaterials.2022.121363. 35063741 PMC7613911

[ref23] Gunawan R. C.; King J. A.; Lee B. P.; Messersmith P. B.; Miller W. M. Surface Presentation of Bioactive Ligands in a Nonadhesive Background Using DOPA-Tethered Biotinylated Poly(Ethylene Glycol). Langmuir 2007, 23 (21), 10635–10643. 10.1021/la701415z. 17803326 PMC2547987

[ref24] Sefkow-Werner J.; Machillot P.; Sales A.; Castro-Ramirez E.; Degardin M.; Boturyn D.; Cavalcanti-Adam E. A.; Albiges-Rizo C.; Picart C.; Migliorini E. Heparan Sulfate Co-Immobilized with CRGD Ligands and BMP2 on Biomimetic Platforms Promotes BMP2-Mediated Osteogenic Differentiation. Acta Biomater. 2020, 114, 90–103. 10.1016/j.actbio.2020.07.015. 32673751

[ref25] Pike D. B.; Cai S.; Pomraning K. R.; Firpo M. A.; Fisher R. J.; Shu X. Z.; Prestwich G. D.; Peattie R. A. Heparin-Regulated Release of Growth Factors in Vitro and Angiogenic Response in Vivo to Implanted Hyaluronan Hydrogels Containing VEGF and BFGF. Biomaterials 2006, 27 (30), 5242–5251. 10.1016/j.biomaterials.2006.05.018. 16806456

[ref26] Jha A. K.; Tharp K. M.; Ye J.; Santiago-Ortiz J. L.; Jackson W. M.; Stahl A.; Schaffer D. V.; Yeghiazarians Y.; Healy K. E. Enhanced Survival and Engraftment of Transplanted Stem Cells Using Growth Factor Sequestering Hydrogels. Biomaterials 2015, 47, 1–12. 10.1016/j.biomaterials.2014.12.043. 25682155 PMC4457539

[ref27] Kirker K. R.; Luo Y.; Nielson J. H.; Shelby J.; Prestwich G. D. Glycosaminoglycan Hydrogel Films as Bio-Interactive Dressings for Wound Healing. Biomaterials 2002, 23 (17), 3661–3671. 10.1016/S0142-9612(02)00100-X. 12109692

[ref28] Cai S.; Liu Y.; Xiao Z. S.; Prestwich G. D. Injectable Glycosaminoglycan Hydrogels for Controlled Release of Human Basic Fibroblast Growth Factor. Biomaterials 2005, 26 (30), 6054–6067. 10.1016/j.biomaterials.2005.03.012. 15958243

[ref29] Patel S.; Kurpinski K.; Quigley R.; Gao H.; Hsiao B. S.; Poo M. M.; Li S. Bioactive Nanofibers: Synergistic Effects of Nanotopography and Chemical Signaling on Cell Guidance. Nano Lett. 2007, 7 (7), 2122–2128. 10.1021/nl071182z. 17567179

[ref30] Le Pennec J.; Picart C.; Vivès R. R.; Migliorini E. Sweet but Challenging: Tackling the Complexity of GAGs with Engineered Tailor-Made Biomaterials. Adv. Mater. 2024, 36 (11), 2312154 10.1002/adma.202312154. 38011916

[ref31] Ladmiral V.; Melia E.; Haddleton D. M. Synthetic Glycopolymers: An Overview. Eur. Polym. J. 2004, 40, 431–449. 10.1016/j.eurpolymj.2003.10.019.

[ref32] Yamaguchi N.; Kiick K. L. Polysaccharide-Poly(Ethylene Glycol) Star Copolymer as a Scaffold for the Production of Bioactive Hydrogels. Biomacromolecules 2005, 6 (4), 1921–1930. 10.1021/bm050003+. 16004429 PMC2887734

[ref33] Yamaguchi N.; Zhang L.; Chae B. S.; Palla C. S.; Furst E. M.; Kiick K. L. Growth Factor Mediated Assembly of Cell Receptor-Responsive Hydrogels. J. Am. Chem. Soc. 2007, 129 (11), 3040–3041. 10.1021/ja0680358. 17315874 PMC2606044

[ref34] Smith R. A.; Lu X.; Tan T.; Luo X.; Le B. Q.; Zubkova O. V.; Cool S.; Nurcombe V. A Synthetic Heparan Sulphate Mimetic for Enhancing BMP-2-Mediated Osteogenesis and Bone Regeneration. Cytotherapy 2020, 22 (5), S32 10.1016/j.jcyt.2020.03.017.

[ref35] Grande D.; Baskaran S.; Chaikof E. L. Glycosaminoglycan Mimetic Biomaterials. 2. Alkene- and Acrylate-Derivatized Glycopolymers via Cyanoxyl-Mediated Free-Radical Polymerization. Macromolecules 2001, 34 (6), 1640–1646. 10.1021/ma001680t.

[ref36] Grande D.; Baskaran S.; Baskaran C.; Gnanou Y.; Chaikof E. L. Glycosaminoglycan-Mimetic Biomaterials. 1. Nonsulfated and Sulfated Glycopolymers by Cyanoxyl-Mediated Free-Radical Polymerization. Macromolecules 2000, 33 (4), 1123–1125. 10.1021/ma991579s.

[ref37] Miura Y. Synthesis and Biological Application of Glycopolymers. J. Polym. Sci., Part A: Polym. Chem. 2007, 45 (22), 5031–5036. 10.1002/pola.22369.

[ref38] Paluck S. J.; Nguyen T. H.; Lee J. P.; Maynard H. D. A Heparin-Mimicking Block Copolymer Both Stabilizes and Increases the Activity of Fibroblast Growth Factor 2 (FGF2). Biomacromolecules 2016, 17 (10), 3386–3395. 10.1021/acs.biomac.6b01182. 27580376 PMC5059753

[ref39] Kolodziej C. M.; Kim S. H.; Broyer R. M.; Saxer S. S.; Decker C. G.; Maynard H. D. Combination of Integrin-Binding Peptide and Growth Factor Promotes Cell Adhesion on Electron-Beam-Fabricated Patterns. J. Am. Chem. Soc. 2012, 134 (1), 247–255. 10.1021/ja205524x. 22126191

[ref40] Christman K. L.; Vázquez-Dorbatt V.; Schopf E.; Kolodziej C. M.; Li R. C.; Broyer R. M.; Chen Y.; Maynard H. D. Nanoscale Growth Factor Patterns by Immobilization on a Heparin-Mimicking Polymer. J. Am. Chem. Soc. 2008, 130 (49), 16585–16591. 10.1021/ja803676r. 19554729 PMC3110987

[ref41] Krishnamoorthy M.; Hakobyan S.; Ramstedt M.; Gautrot J. E. Surface-Initiated Polymer Brushes in the Biomedical Field: Applications in Membrane Science, Biosensing, Cell Culture, Regenerative Medicine and Antibacterial Coatings. Chem. Rev. 2014, 114 (21), 10976–11026. 10.1021/cr500252u. 25353708

[ref42] Li D.; Xu L.; Wang J.; Gautrot J. E. Responsive Polymer Brush Design and Emerging Applications for Nanotheranostics. Adv. Healthcare Mater. 2021, 10, 2000953 10.1002/adhm.202000953. PMC11468394 32893474

[ref43] Neri-Cruz C. E.; Teixeira F. M. E.; Gautrot J. E. A Guide to Functionalisation and Bioconjugation Strategies to Surface-Initiated Polymer Brushes. Chem. Commun. 2023, 59 (49), 7534–7558. 10.1039/D3CC01082A. PMC10270241 37194961

[ref44] Zoppe J. O.; Ataman N. C.; Mocny P.; Wang J.; Moraes J.; Klok H. A. Surface-Initiated Controlled Radical Polymerization: State-of-the-Art, Opportunities, and Challenges in Surface and Interface Engineering with Polymer Brushes. Chem. Rev. 2017, 117 (3), 1105–1318. 10.1021/acs.chemrev.6b00314. 28135076

[ref45] Rodriguez Emmenegger C.; Brynda E.; Riedel T.; Sedlakova Z.; Houska M.; Alles A. B. Interaction of Blood Plasma with Antifouling Surfaces. Langmuir 2009, 25 (11), 6328–6333. 10.1021/la900083s. 19408903

[ref46] Ladd J.; Zhang Z.; Chen S.; Hower J. C.; Jiang S. Zwitterionic Polymers Exhibiting High Resistance to Nonspecific Protein Adsorption from Human Serum and Plasma. Biomacromolecules 2008, 9 (5), 1357–1361. 10.1021/bm701301s. 18376858

[ref47] Rodriguez-Emmenegger C.; Brynda E.; Riedel T.; Houska M.; Šubr V.; Alles A. B.; Hasan E.; Gautrot J. E.; Huck W. T. S. Polymer Brushes Showing Non-Fouling in Blood Plasma Challenge the Currently Accepted Design of Protein Resistant Surfaces. Macromol. Rapid Commun. 2011, 32 (13), 952–957. 10.1002/marc.201100189. 21644241

[ref48] Desseaux S.; Klok H. A. Fibroblast Adhesion on ECM-Derived Peptide Modified Poly(2-Hydroxyethyl Methacrylate) Brushes: Ligand Co-Presentation and 3D-Localization. Biomaterials 2015, 44, 24–35. 10.1016/j.biomaterials.2014.12.011. 25617123

[ref49] Tugulu S.; Silacci P.; Stergiopulos N.; Klok H. A. RGD-Functionalized Polymer Brushes as Substrates for the Integrin Specific Adhesion of Human Umbilical Vein Endothelial Cells. Biomaterials 2007, 28 (16), 2536–2546. 10.1016/j.biomaterials.2007.02.006. 17321591

[ref50] Colak B.; Di Cio S.; Gautrot J. E. Biofunctionalized Patterned Polymer Brushes via Thiol-Ene Coupling for the Control of Cell Adhesion and the Formation of Cell Arrays. Biomacromolecules 2018, 19 (5), 1445–1455. 10.1021/acs.biomac.7b01436. 29294284

[ref51] Tang Z.; Okano T. Recent Development of Temperature-Responsive Surfaces and Their Application for Cell Sheet Engineering. Regener. Biomater. 2014, 1, 91–102. 10.1093/rb/rbu011. PMC4669004 26816628

[ref52] Akiyama Y.; Kikuchi A.; Yamato M.; Okano T. Ultrathin Poly(N-Isopropylacrylamide) Grafted Layer on Polystyrene Surfaces for Cell Adhesion/Detachment Control. Langmuir 2004, 20 (13), 5506–5511. 10.1021/la036139f. 15986693

[ref53] Li D.; Sharili A. S.; Connelly J.; Gautrot J. E. Highly Stable RNA Capture by Dense Cationic Polymer Brushes for the Design of Cytocompatible, Serum-Stable SiRNA Delivery Vectors. Biomacromolecules 2018, 19 (2), 606–615. 10.1021/acs.biomac.7b01686. 29338211

[ref54] Qu F.; Li D.; Ma X.; Chen F.; Gautrot J. E. A Kinetic Model of Oligonucleotide-Brush Interactions for the Rational Design of Gene Delivery Vectors. Biomacromolecules 2019, 20 (6), 2218–2229. 10.1021/acs.biomac.9b00155. 31017767

[ref55] Raynold A. A. M.; Li D.; Chang L.; Gautrot J. E. Competitive Binding and Molecular Crowding Regulate the Cytoplasmic Interactome of Non-Viral Polymeric Gene Delivery Vectors. Nat. Commun. 2021, 12 (1), 6445 10.1038/s41467-021-26695-w. 34750370 PMC8576037

[ref56] De Vos W. M.; Biesheuvel P. M.; De Keizer A.; Kleijn J. M.; Stuart M. A. C. Adsorption of the Protein Bovine Serum Albumin in a Planar Poly(Acrylic Acid) Brush Layer as Measured by Optical Reflectometry. Langmuir 2008, 24 (13), 6575–6584. 10.1021/la8006469. 18507422

[ref57] Henzler K.; Haupt B.; Lauterbach K.; Wittemann A.; Borisov O.; Ballauff M. Adsorption of β-Lactoglobulin on Spherical Polyelectrolyte Brushes: Direct Proof of Counterion Release by Isothermal Titration Calorimetry. J. Am. Chem. Soc. 2010, 132 (9), 3159–3163. 10.1021/ja909938c. 20143809

[ref58] Psarra E.; Foster E.; König U.; You J.; Ueda Y.; Eichhorn K. J.; Müller M.; Stamm M.; Revzin A.; Uhlmann P. Growth Factor-Bearing Polymer Brushes - Versatile Bioactive Substrates Influencing Cell Response. Biomacromolecules 2015, 16 (11), 3530–3542. 10.1021/acs.biomac.5b00967. 26447354

[ref59] Liao S.; He Q.; Yang L.; Liu S.; Zhang Z.; Guidoin R.; Fu Q.; Xie X. Toward Endothelialization via Vascular Endothelial Growth Factor Immobilization on Cell-Repelling Functional Polyurethanes. J. Biomed. Mater. Res., Part B 2019, 107 (4), 965–977. 10.1002/jbm.b.34190. 30265778

[ref60] Yang E. Y.; Kronenfeld J. P.; Gattás-Asfura K. M.; Bayer A. L.; Stabler C. L. Engineering an “infectious” Treg biomimetic through chemoselective tethering of TGF-β1 to PEG brush surfaces. Biomaterials 2015, 67, 20–31. 10.1016/j.biomaterials.2015.07.009. 26197412 PMC4550500

[ref61] Di Luca A.; Klein-Gunnewiek M.; Vancso J. G.; van Blitterswijk C. A.; Benetti E. M.; Moroni L. Covalent Binding of Bone Morphogenetic Protein-2 and Transforming Growth Factor-β3 to 3D Plotted Scaffolds for Osteochondral Tissue Regeneration. Biotechnol. J. 2017, 12 (12), 1700072 10.1002/biot.201700072. 28865136

[ref62] Gan Q.; Chen L.; Bei H. P.; Ng S. W.; Guo H.; Liu G.; Pan H.; Liu C.; Zhao X.; Zheng Z. Artificial Cilia for Soft and Stable Surface Covalent Immobilization of Bone Morphogenetic Protein-2. Bioact. Mater. 2023, 24, 551–562. 10.1016/j.bioactmat.2022.12.029. 36714333 PMC9845954

[ref63] Arisaka Y.; Kobayashi J.; Yamato M.; Akiyama Y.; Okano T. Switching of Cell Growth/Detachment on Heparin-Functionalized Thermoresponsive Surface for Rapid Cell Sheet Fabrication and Manipulation. Biomaterials 2013, 34 (17), 4214–4222. 10.1016/j.biomaterials.2013.02.056. 23498894

[ref64] Kobayashi J.; Arisaka Y.; Yui N.; Yamato M.; Okano T. Preservation of Heparin-Binding EGF-like Growth Factor Activity on Heparin-Modified Poly(: N -Isopropylacrylamide)-Grafted Surfaces. RSC Adv. 2021, 11 (59), 37225–37232. 10.1039/D1RA07317F. 35496401 PMC9043771

[ref65] Ayres N.; Holt D. J.; Jones C. F.; Corum L. E.; Grainger D. W. Polymer Brushes Containing Sulfonated Sugar Repeat Units: Synthesis, Characterization, and in Vitro Testing of Blood Coagulation Activation. J. Polym. Sci., Part A: Polym. Chem. 2008, 46 (23), 7713–7724. 10.1002/pola.23075. PMC2766538 19859552

[ref66] Isahak N.; Sanchez J.; Perrier S.; Stone M. J.; Payne R. J. Synthesis of Polymers and Nanoparticles Bearing Polystyrene Sulfonate Brushes for Chemokine Binding. Org. Biomol. Chem. 2016, 14 (24), 5652–5658. 10.1039/C6OB00270F. 27031327

[ref67] Henzler K.; Haupt B.; Rosenfeldt S.; Harnau L.; Narayanan T.; Ballauff M. Interaction Strength between Proteins and Polyelectrolyte Brushes: A Small Angle X-Ray Scattering Study. Phys. Chem. Chem. Phys. 2011, 13 (39), 17599–17605. 10.1039/c1cp20663j. 21892474

[ref68] Haupt B.; Neumann T.; Wittemann A.; Ballauff M. Activity of Enzymes Immobilized in Colloidal Spherical Polyelectrolyte Brushes. Biomacromolecules 2005, 6 (2), 948–955. 10.1021/bm0493584. 15762664

[ref69] Rzhepishevska O.; Hakobyan S.; Ruhal R.; Gautrot J.; Barbero D.; Ramstedt M. The Surface Charge of Anti-Bacterial Coatings Alters Motility and Biofilm Architecture. Biomater. Sci. 2013, 1 (6), 589–602. 10.1039/c3bm00197k. 32481834

[ref70] Ramstedt M.; Cheng N.; Azzaroni O.; Mossialos D.; Mathieu H. J.; Huck W. T. S. Synthesis and Characterization of Poly(3-Sulfopropylmethacrylate) Brushes for Potential Antibacterial Applications. Langmuir 2007, 23 (6), 3314–3321. 10.1021/la062670+. 17291016

[ref71] Jones D. M.; Brown A. A.; Huck W. T. S. Surface-Initiated Polymerizations in Aqueous Media: Effect of Initiator Density. Langmuir 2002, 18 (4), 1265–1269. 10.1021/la011365f.

[ref72] Krishnamoorthy M.; Li D.; Sharili A. S.; Gulin-Sarfraz T.; Rosenholm J. M.; Gautrot J. E. Solution Conformation of Polymer Brushes Determines Their Interactions with DNA and Transfection Efficiency. Biomacromolecules 2017, 18 (12), 4121–4132. 10.1021/acs.biomac.7b01175. 29020443

[ref73] Trmcic-Cvitas J.; Hasan E.; Ramstedt M.; Li X.; Cooper M. A.; Abell C.; Huck W. T. S.; Gautrot J. E. Biofunctionalized Protein Resistant Oligo(Ethylene Glycol)-Derived Polymer Brushes as Selective Immobilization and Sensing Platforms. Biomacromolecules 2009, 10 (10), 2885–2894. 10.1021/bm900706r. 19761181

[ref74] Stephens P.; Grenard P.; Aeschlimann P.; Langley M.; Blain E.; Errington R.; Kipling D.; Thomas D.; Aeschlimann D. Crosslinking and G-Protein Functions of Transglutaminase 2 Contribute Differentially to Fibroblast Wound Healing Responses. J. Cell Sci. 2004, 117, 3389–3403. 10.1242/jcs.01188. 15199098

[ref75] Pan X.; Fantin M.; Yuan F.; Matyjaszewski K. Externally Controlled Atom Transfer Radical Polymerization. Chem. Soc. Rev. 2018, 47 (14), 5457–5490. 10.1039/C8CS00259B. 29868657

[ref76] Paniagua S. A.; Kim Y.; Henry K.; Kumar R.; Perry J. W.; Marder S. R. Surface-Initiated Polymerization from Barium Titanate Nanoparticles for Hybrid Dielectric Capacitors. ACS Appl. Mater. Interfaces 2014, 6 (5), 3477–3482. 10.1021/am4056276. 24490753

[ref77] Hansson S.; Östmark E.; Carlmark A.; Malmström E. ARGET ATRP for Versatile Grafting of Cellulose Using Various Monomers. ACS Appl. Mater. Interfaces 2009, 1 (11), 2651–2659. 10.1021/am900547g. 20356139

[ref78] Hackett A. J.; Malmström J.; Molino P. J.; Gautrot J. E.; Zhang H.; Higgins M. J.; Wallace G. G.; Williams D. E.; Travas-Sejdic J. Conductive Surfaces with Dynamic Switching in Response to Temperature and Salt. J. Mater. Chem. B 2015, 3 (48), 9285–9294. 10.1039/c5tb02125a. 32262927

[ref79] Cheesman B. T.; Neilson A. J. G.; Willott J. D.; Webber G. B.; Edmondson S.; Wanless E. J. Effect of Colloidal Substrate Curvature on PH-Responsive Polyelectrolyte Brush Growth. Langmuir 2013, 29 (20), 6131–6140. 10.1021/la4004092. 23617419

[ref80] Pavlovic E.; Quist A. P.; Gelius U.; Nyholm L.; Oscarsson S. Generation of Thiolsulfinates/Thiolsulfonates by Electrooxidation of Thiols on Silicon Surfaces for Reversible Immobilization of Molecules. Langmuir 2003, 19 (10), 4217–4221. 10.1021/la026846t.

[ref81] Tan K. Y.; Lin H.; Ramstedt M.; Watt F. M.; Huck W. T. S.; Gautrot J. E. Decoupling Geometrical and Chemical Cues Directing Epidermal Stem Cell Fate on Polymer Brush-Based Cell Micro-Patterns. Integr. Biol. 2013, 5 (6), 899–910. 10.1039/c3ib40026c. 23572192

[ref82] He G. L.; Merlitz H.; Sommer J. U.; Wu C. X. Static and Dynamic Properties of Polymer Brushes at Moderate and High Grafting Densities: A Molecular Dynamics Study. Macromolecules 2007, 40 (18), 6721–6730. 10.1021/ma070983l.

[ref83] De Vos W. M.; Leermakers F. A. M.; De Keizer A.; Mieke Kleijn J.; Cohen Stuart M. A. Interaction of Particles with a Polydisperse Brush: A Self-Consistent-Field Analysis. Macromolecules 2009, 42 (15), 5881–5891. 10.1021/ma900819b.

[ref84] Milner S. T.; Witten T. A.; Cates M. E. Effects of Polydispersity in the End-Grafted Polymer Brush. Macromolecules 1989, 22 (2), 853–861. 10.1021/ma00192a057.

[ref85] De Vos W. M.; Leermakers F. A. M.; De Keizer A.; Stuart M. A. C.; Kleijn J. M. Field Theoretical Analysis of Driving Forces for the Uptake of Proteins by Like-Charged Polyelectrolyte Brushes: Effects of Charge Regulation and Patchiness. Langmuir 2010, 26 (1), 249–259. 10.1021/la902079u. 19697905

[ref86] Becker A. L.; Welsch N.; Schneider C.; Ballauff M. Adsorption of RNase A on Cationic Polyelectrolyte Brushes: A Study by Isothermal Titration Calorimetry. Biomacromolecules 2011, 12 (11), 3936–3944. 10.1021/bm200954j. 21970466

[ref87] Gautrot J. E.; Chang L.; Alexis C.; Gutfreund P.; Zarbakhsh A. The Architecture of Oligonucleotide-Polycationic Brush Complexes - A Neutron Reflectometry Study. Adv. Mater. Interfaces 2022, 9 (33), 2201344 10.1002/admi.202201344.

[ref88] Le Pennec J.; Guibert A.; Vives R. R.; Migliorini E. BMP2 Binds Non-Specifically to PEG-Passivated Biomaterials and Induces Substantial Signaling. BioRxiv 2024, 03.14.585026. 10.1002/mabi.202400169 39215622

[ref89] Botchkarev V. A. Bone Morphogenetic Proteins and Their Antagonists in Skin and Hair Follicle Biology. J. Invest. Dermatol. 2003, 120, 36–47. 10.1046/j.1523-1747.2003.12002.x. 12535196

[ref90] Cai H.; Zou J.; Wang W.; Yang A. BMP2 Induces HMSC Osteogenesis and Matrix Remodeling. Mol. Med. Rep. 2020, 23 (2), 125 10.3892/mmr.2020.11764. 33300084 PMC7751477

[ref91] Davidson E. N. B.; Vitters E. L.; van Lent P. L. E. M.; van de Loo F. A. J.; van den Berg W. B.; van der Kraan P. M. Elevated Extracellular Matrix Production and Degradation upon Bone Morphogenetic Protein-2 (BMP-2) Stimulation Point toward a Role for BMP-2 in Cartilage Repair and Remodeling. Arthritis Res. Ther. 2007, 9 (5), R102 10.1186/ar2305. 17922907 PMC2212581

[ref92] Di Cio S.; Gautrot J. E. Cell Sensing of Physical Properties at the Nanoscale: Mechanisms and Control of Cell Adhesion and Phenotype. Acta Biomater. 2016, 30, 26–48. 10.1016/j.actbio.2015.11.027. 26596568

[ref93] Hattori S.; Adachi E.; Ebihara T.; Shirai T.; Someki I.; Irie S. Alkali-Treated Collagen Retained the Triple Helical Conformation and the Ligand Activity for the Cell Adhesion via 2 1 Integrin. J. Biochem. 1999, 125 (4), 676–684. 10.1093/oxfordjournals.jbchem.a022336. 10101279

[ref94] Highberger J. H. The Isoelectric Point of Collagen. J. Am. Chem. Soc. 1939, 61 (9), 2302–2303. 10.1021/ja01878a010.

[ref95] Martino M. M.; Hubbell J. A. The 12th-14th Type III Repeats of Fibronectin Function as a Highly Promiscuous Growth Factor-binding Domain. FASEB J. 2010, 24 (12), 4711–4721. 10.1096/fj.09.151282. 20671107

[ref96] Graham J.; Raghunath M.; Vogel V. Fibrillar Fibronectin Plays a Key Role as Nucleator of Collagen i Polymerization during Macromolecular Crowding-Enhanced Matrix Assembly. Biomater. Sci. 2019, 7 (11), 4519–4535. 10.1039/C9BM00868C. 31436263 PMC6810780

[ref97] Nashchekina Y.; Nikonov P.; Prasolov N.; Sulatsky M.; Chabina A.; Nashchekin A. The Structural Interactions of Molecular and Fibrillar Collagen Type I with Fibronectin and Its Role in the Regulation of Mesenchymal Stem Cell Morphology and Functional Activity. Int. J. Mol. Sci. 2022, 23 (20), 12577 10.3390/ijms232012577. 36293432 PMC9604100

[ref98] Paul J. I.; Hynes R. O. Multiple Fibronectin Subunits and Their Post-Translational Modifications. J. Biol. Chem. 1984, 259 (21), 13477–13487. 10.1016/S0021-9258(18)90719-2. 6490662

[ref99] Cardin A. D.; Weintraub H. J. R. Molecular Modeling of Protein-Glycosaminoglycan Interactions. Arteriosclerosis 1989, 9 (1), 21–32. 10.1161/01.ATV.9.1.21. 2463827

[ref100] Ruppert R.; Hoffmann E.; Sebald W. Human Bone Morphogenetic Protein 2 Contains a Heparin-Binding Site Which Modifies Its Biological Activity. Eur. J. Biochem. 1996, 237 (1), 295–302. 10.1111/j.1432-1033.1996.0295n.x. 8620887

[ref101] Le Pennec J.; Makshakova O.; Nevola P.; Fouladkar F.; Gout E.; Machillot P.; Friedel-Arboleas M.; Picart C.; Perez S.; Vortkamp A.; Vives R. R.; Migliorini E. Glycosaminoglycans exhibit distinct interactions and signaling with BMP2 according to their nature and localization. Carbohydrate Polymers 2024, 341, 122294 10.1016/j.carbpol.2024.122294. 38876708

