# Correction to “BMP-Binding Polysulfonate Brushes to Control Growth Factor Presentation and Regulate Matrix Remodelling”

**DOI:** 10.1021/acsami.5c12879

**Published:** 2025-07-10

**Authors:** Metzli Hernandez Marchena, Elisa Lambert, Bojana Bogdanović, Fauzia Quadir, Carlos E. Neri-Cruz, Jiajun Luo, Clemence Nadal, Elisa Migliorini, Julien E. Gautrot

In the Supporting Information of the original article, some of the images displayed in Figure S12 were duplicated by mistake during formatting of the figure. These correspond to PSPMA-LD10/PDPMA/Day5 and PSPMA-LD10/PDPMA-BMP2/Day7. The correct images are shown in the corrected Supporting Information attached. In addition, in Figure S15, some of the labels describing the conditions associated with images presented were inverted by mistake during formatting. Specifically, “PSPMA” and “PSPMA-BMP2” labels were inverted for all images, and “PSPMA-LD10” and “PSPMA-HD10” were inverted. The correct labeling is shown in the corrected Supporting Information. These changes do not effect the scientific conclusions of the paper.

## Supplementary Material



## Notes

Additional ellipsometry, XPS, FTIR, SPR, QCM, SEM, AFM, and microscopy data (PDF)

