# Supplementary material for: BMP-Binding Polysulfonate Brushes to Control Growth Factor Presentation and Regulate Matrix Remodelling
Source: ACS Appl Mater Interfaces. 2024 Jul 29;16(31):40455–68. doi: 10.1021/acsami.4c05139 (PMC11310902; doi:10.1021/acsami.4c05139)
Supplement: Supplementary file 1 — am4c05139_si_001.pdf [file am4c05139_si_001.pdf]

BMP-Binding Polysulfonate Brushes to Control Growth Factor Presentation and Regulate Matrix Remodelling

Supporting Information

#Metzli Hernandez Marchena[1], #Elisa Lambert[2], Bojana Bogdanović[2], Fauzia Quadir[1], Carlos E. Neri-Cruz[1], Jiajun Luo[1], Clemence Nadal[1], Elisa Migliorini[2*] and Julien E. Gautrot[1*]

[1] School of Engineering and Materials Science, Queen Mary University of London, Mile End Road, London E1 4NS, United Kingdom.

[2] University Grenoble Alpes, INSERM, CEA, CNRS, U1292 Biosanté, EMR 5000, 17 Av des martyrs, 38000 Grenoble, France.

* Correspondence:

Elisa Migliorini (elisa.migliorini@cea.fr)

Julien E. Gautrot (j.gautrot@qmul.ac.uk)

# M.H.M. and E.L. contributed equally to this work.

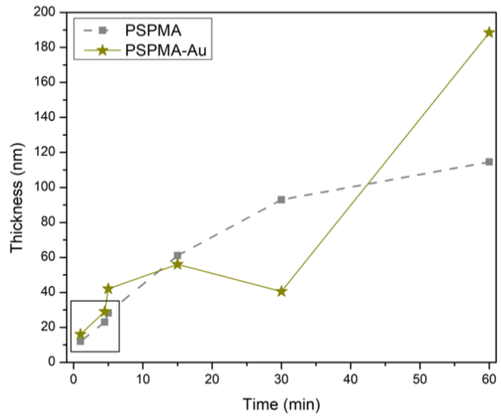

Figure S1. Growth of PSPMA brushes via ARGET from silicon and gold substrates (thiol monolayers). Brown: PSPMA on gold surfaces, Gray: PSPMA on silicon surfaces.

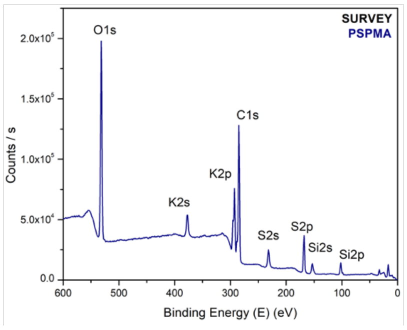

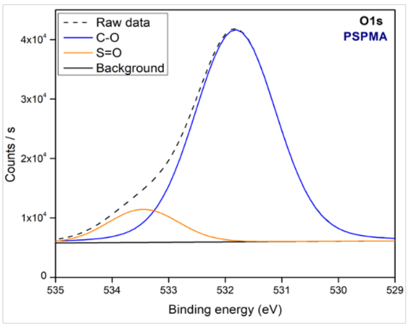

Element	Atomic %
C	44.9
S	7.37
O	36.0
K	6.28
Si	5.41

Figure S2. XPS Survey spectrum of PSPMA (left), high-resolution elemental scan of O1s (middle). Table reports the atomic composition PSPMA (right).

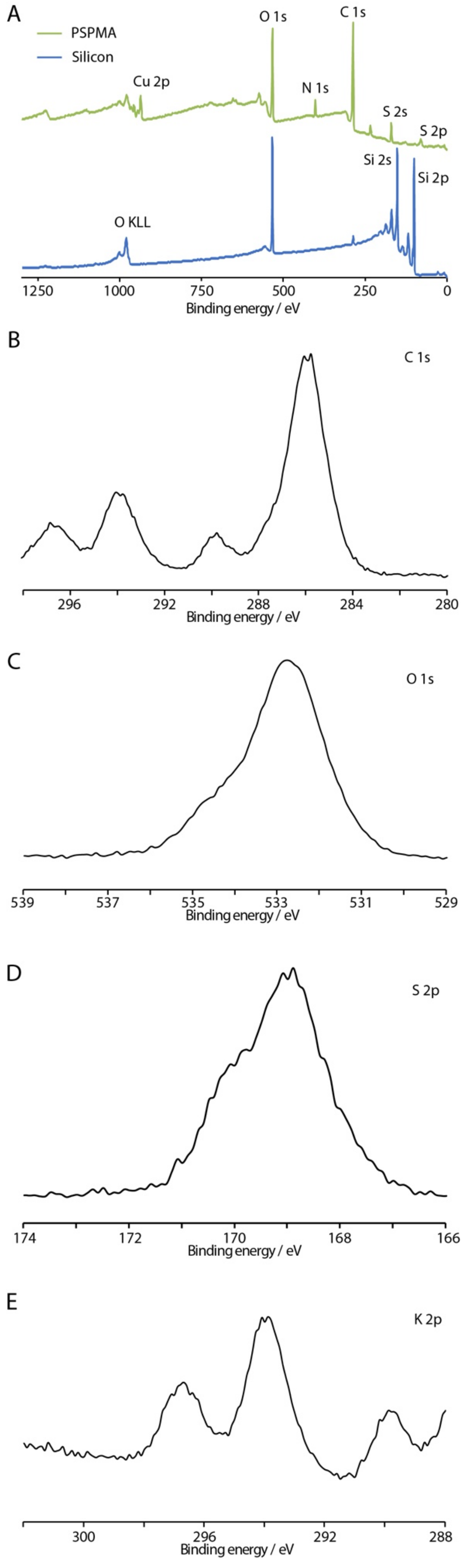

Figure S3. (A) XPS full spectrum surveys of silicon substrates and PSPMA brushes generated via ATRP. High resolution spectra of C 1s (B), O 1s (C), S 2p (D) and K 2p (E) regions.

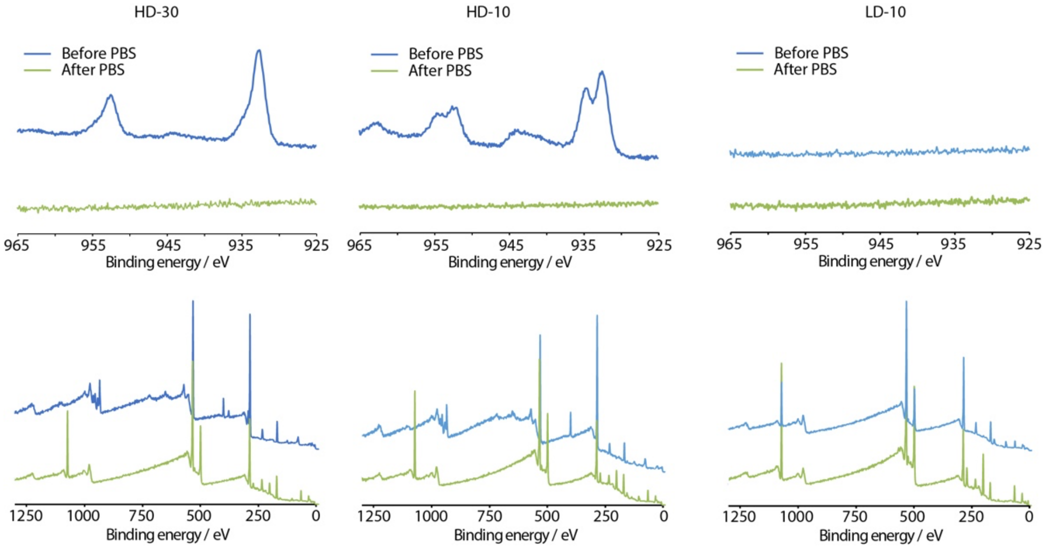

Figure S4. XPS spectra at different densities and thickness of PSPMA brushes before and after 1 h incubation in buffer (PBS). Top, Cu 2p high resolution spectrum. Bottom, full range XPS survey.

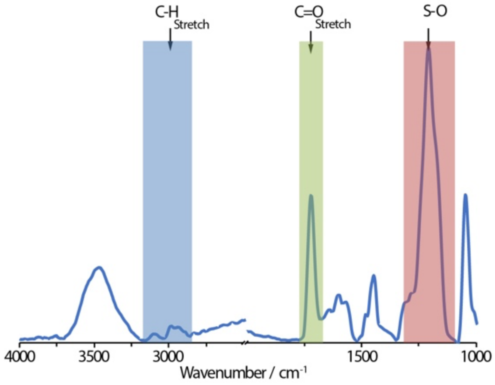

Figure S5. Grazing angle FTIR spectrum of a poly-3-sulfopropyl methacrylate (PSPMA) brush generated via ATRP. Characteristic functional groups are highlighted in blue, green and red.

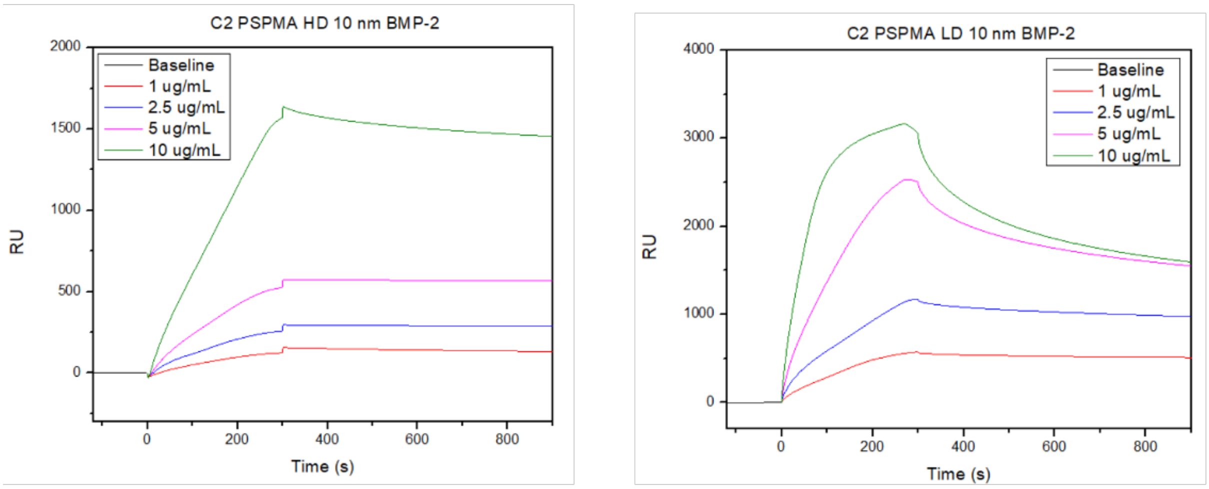

Figure S6. SPR traces data for the binding of BMP-2, at different concentrations, to dense (left) and sparse (right) 10 nm PSPMA brushes generated by ARGET.

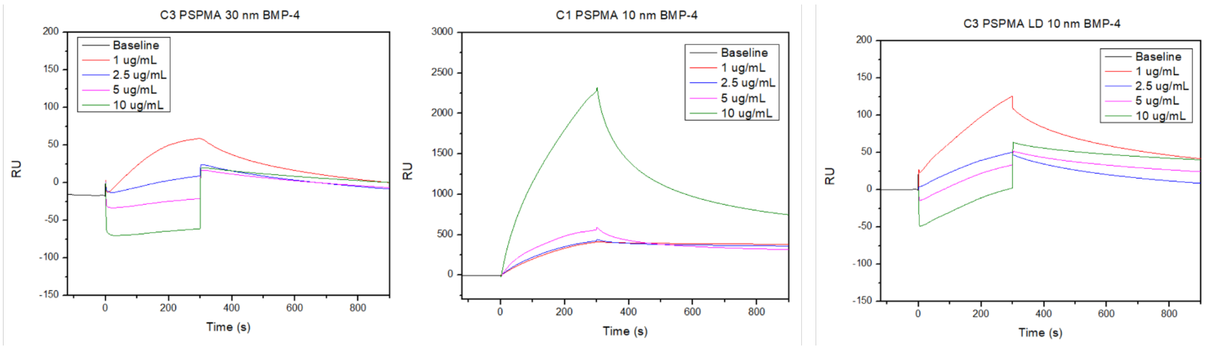

Figure S7. SPR traces data for the binding of BMP-4, at different concentrations to PSPMA brushes (from ARGET). HD PSPMA 30 nm (left), HD PSPMA 10 nm (middle), LD PSPMA 10 nm (right).

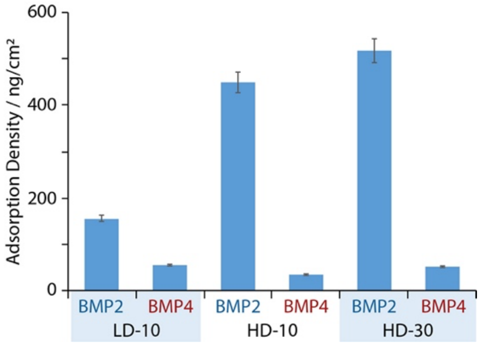

Figure S8. Summary of the adsorption density of BMP2/BMP4 to PSPMA brushes of different densities via ATRP (from 10 µg/mL BMP solutions).

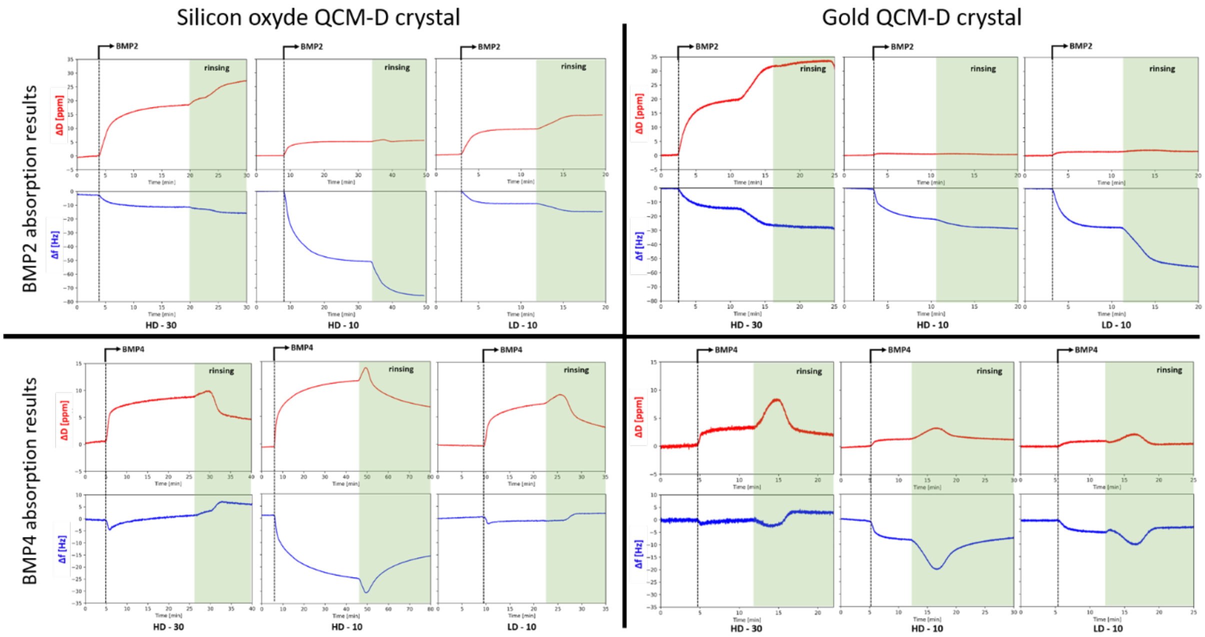

Figure S9. QCM-D data showing the binding of BMP2 and BMP4 (10 μg/mL) to thick and dense brushes HD30, thin and dense (HD 10) and thin and sparse PSPMA brushes (LD 10) grown from gold-coated substrates. There the frequency and the dissipation of the third overtone have been presented

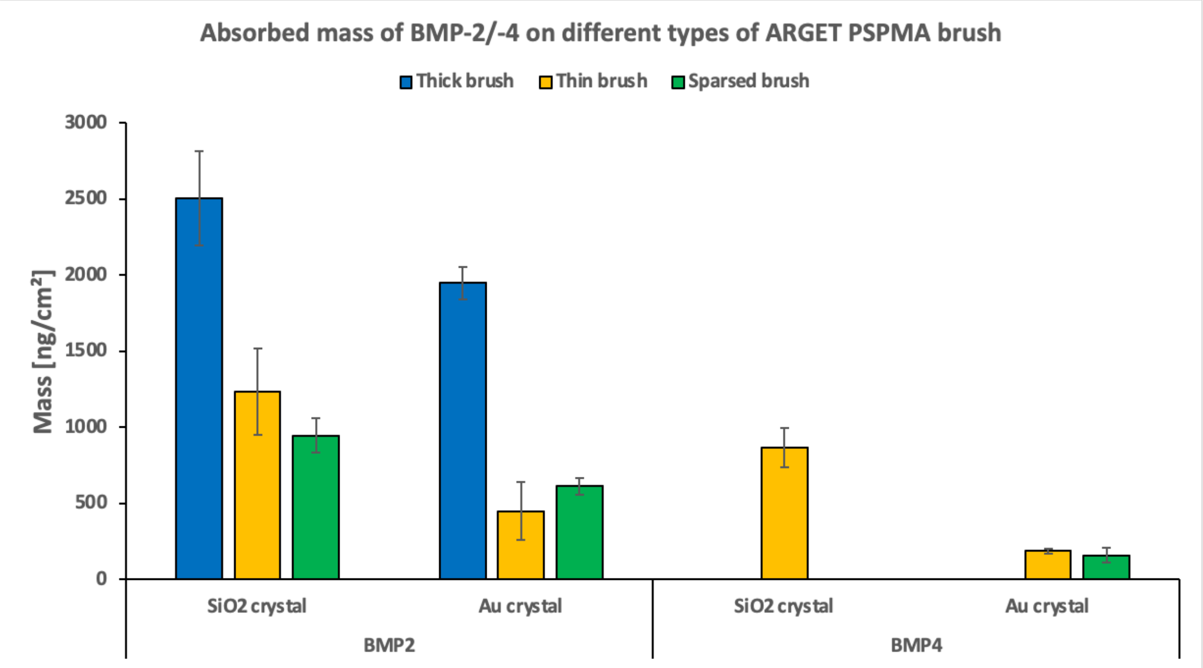

Figure S10. Full summary of BMP2 and BMP4 adsorption (10 μg/mL solutions) on different brushes on Au and on SiO_2 QCMD crystals. Here the areal mass density has been obtained by Dfind software (Biolin scientific) by using the Broadfit model. We can observe that the tendency are comparable, however we observed a higher mass density on SiO_2 surfaces with respect to Au.

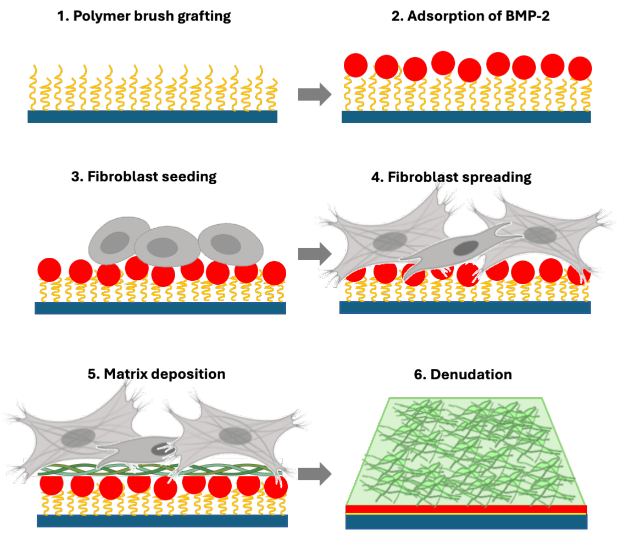

Figure S11. Schematic representation of the different steps associated with the functionalisation of PSPMA brushes with BMP-2, cell seeding and matrix deposition.

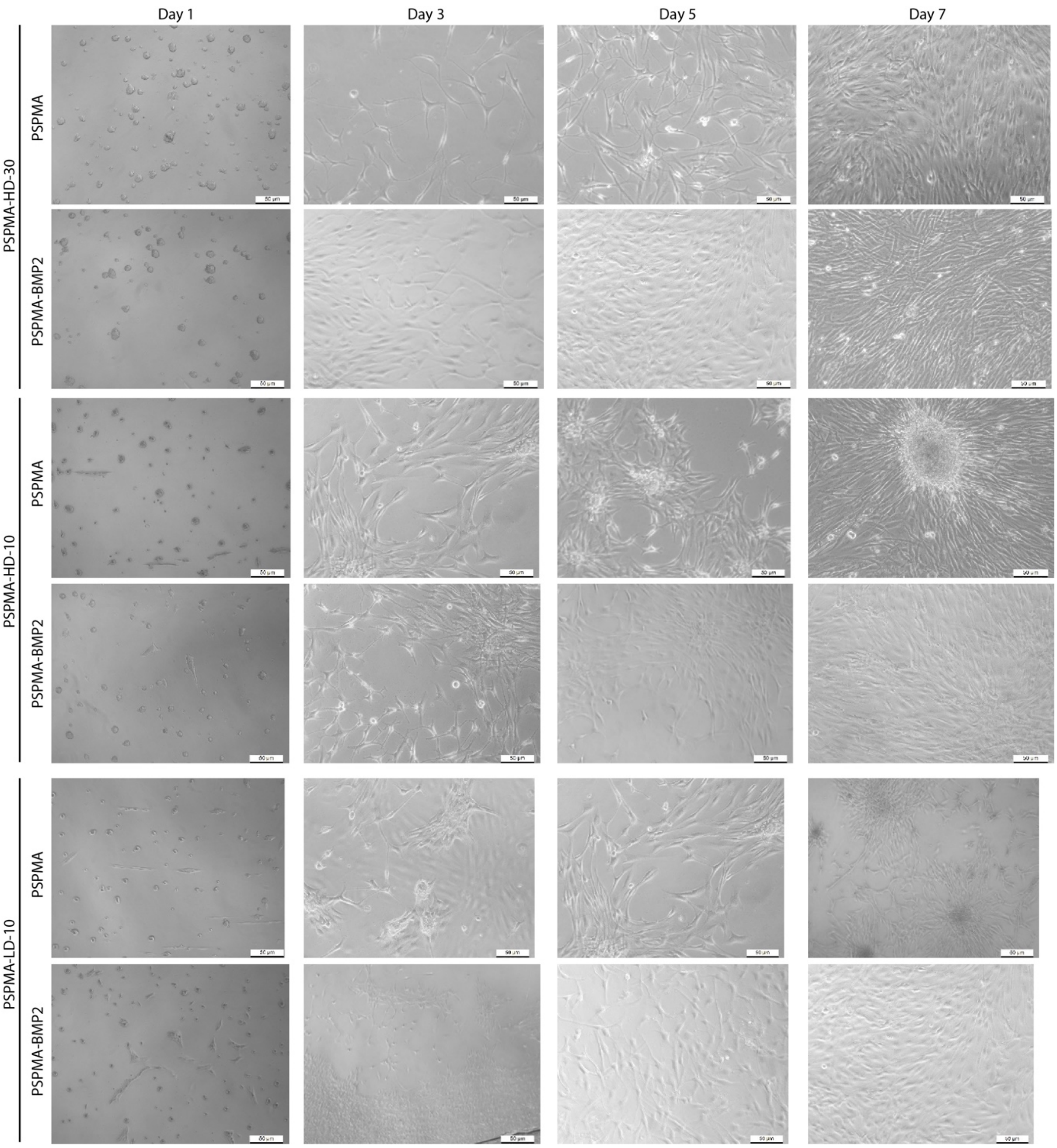

Figure S12. Dermal fibroblasts cultured at the surface of PSPMA brushes (HD-30, HD-10 and LD-10), with and without BMP2 coatings (10 μg/mL), at days 1-7 (bright field microscopy images).

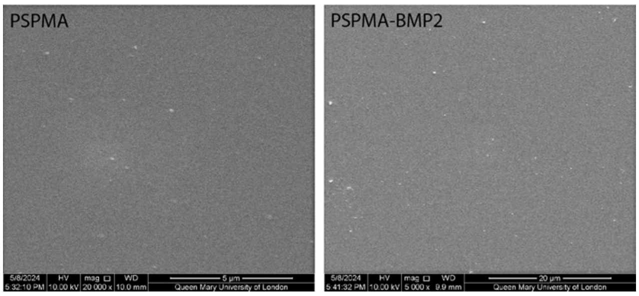

Figure S13. SEM images of dense PSPMA brushes prior and after adsorption of BMP2.

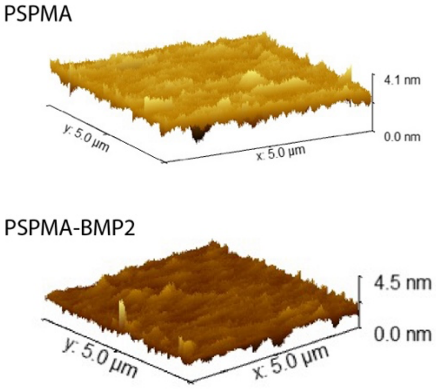

Figure S14. AFM images of dense PSPMA brushes prior and after adsorption of BMP2.

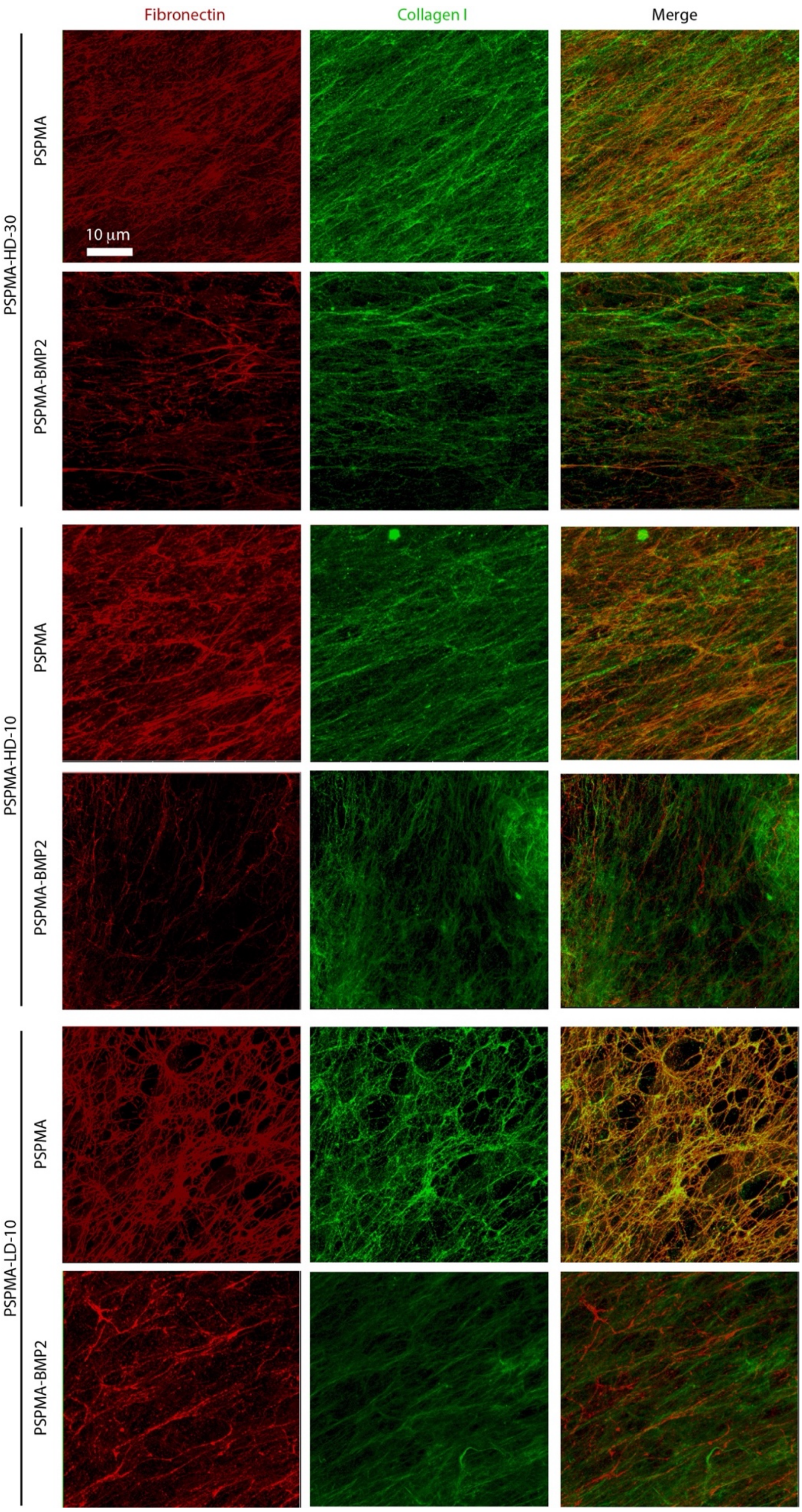

Figure S15. Confocal microscopy images of fibronectin and collagen I fibre mats deposited by dermal fibroblasts cultured for 7 days at the surface of PSPMA brushes (HD-30, HD-10, LD-10) with and without pre-adsorbed BMP2 (10 μg/mL).

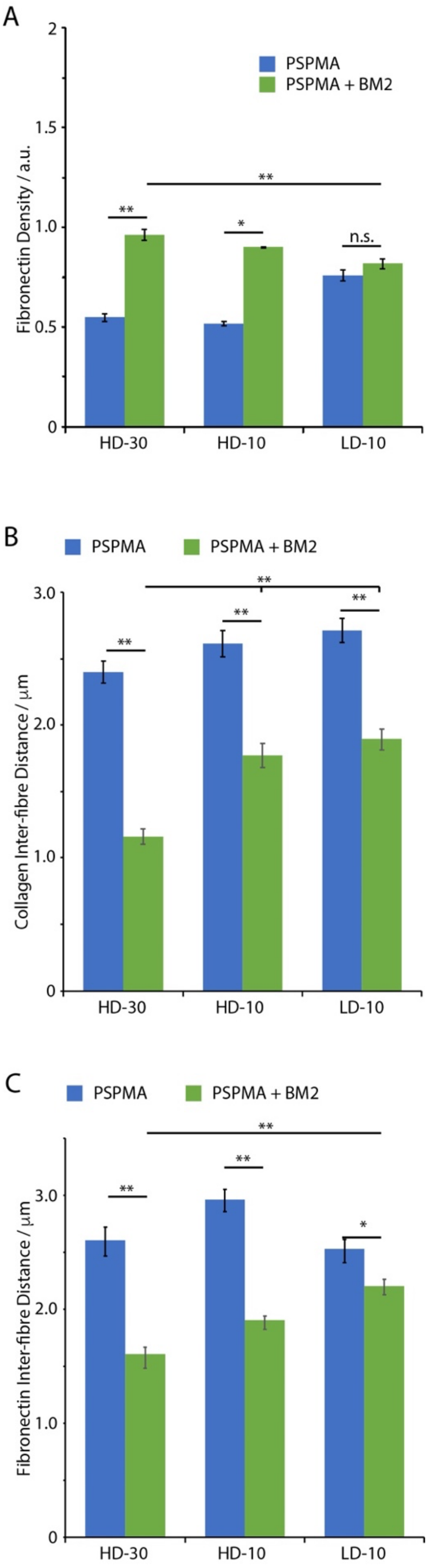

Figure S16. Quantification of the density (A) of fibronectin deposited by dermal fibroblasts, and the inter-fibre spacing measured from confocal microscopy images for collagen I (B) and fibronectin (C) networks deposited by dermal fibroblasts growing at the surface of PSPMA brushes with and without pre-adsorbed BMP2 (10 μg/mL), for 7 days.
